# Bailing capsule (*Cordyceps sinensis*) ameliorates renal triglyceride accumulation through the PPARα pathway in diabetic rats

**DOI:** 10.3389/fphar.2022.915592

**Published:** 2022-08-25

**Authors:** Qian Zhang, Xinhua Xiao, Ming Li, Miao Yu, Fan Ping

**Affiliations:** Key Laboratory of Endocrinology, Department of Endocrinology, Ministry of Health, Peking Union Medical College Hospital, Peking Union Medical College, Chinese Academy of Medical Sciences, Beijing, China

**Keywords:** *Cordyceps sinesis*, diabetic nephropathy, lipid accumulation, transcriptome analysis, PPARα

## Abstract

Diabetic nephropathy (DN) is a severe diabetic complication of the kidney and is the main cause of end-stage kidney disease worldwide. *Cordyceps sinensis* (*C. sinensis*) is not only a traditional Chinese medicine (TCM) but also a healthy food. In China, *C. sinensis* has been widely used to treat various kidney diseases. Bailing Capsule, which active ingredient is *C. sinensis*, is approved to treat kidney disease, respiratory disease, and immune disease. However, its underlying mechanism in DN remains unclear. The purpose of the present study was to investigate the underlying mechanism of Bailing Capsule on kidney in diabetic rats. The DN model was established by streptozotocin (STZ) injection. Low and high doses of Bailing Capsule were orally administrated for 12 weeks after diabetes induction. Renal function was evaluated by serum creatinine, blood urea nitrogen, 24-h urinary protein, and urinary albumin. Mesangial matrix expansion and renal fibrosis were measured using histopathology staining. We found that the disorder of renal function and pathology in DN rats was significantly modified by Bailing Capsule treatment. Consistently, Bailing Capsule markedly alleviated DN rat glomerulosclerosis, tubulointerstitial injury and renal fibrosis as shown by pathological staining. Moreover, Bailing Capsule significantly reduced the kidney triglyceride content and renal lipid droplet formation in DN rats. The renal transcriptome revealed that Bailing Capsule-treated kidneys had 498 upregulated genes and 448 downregulated genes. These differentially expressed genes were enriched in the peroxisome proliferator activated receptor (PPAR) pathway and fatty acid metabolism function ontology. mRNA and protein expression analyses revealed substantial enhancement of the lipolysis pathway and inhibition of lipogenesis in Bailing Capsule-treated rat kidneys compared to DN rats. Bailing Capsule activated the expression of PPARα, ACOX1 (acyl-CoA oxidase 1), and SCD (stearoyl-CoA desaturase) in diabetic nephropathy while suppressing the expression of FASN (fatty acid synthase). In conclusion, Bailing Capsule could attenuate renal triglyceride accumulation in diabetic rats by moderating PPARα pathway.

## Introduction

Diabetes mellitus (DM) is a chronic metabolic disease characterized by hyperglycemia. The main mechanism of DM is insulin deficiency or insulin production and/or action disorder. Currently, DM is trending toward an increasingly severe epidemic worldwide (Zimmet et al., 2016). According to the 10th edition of the International Diabetes Federation (IDF) published in January 2022, the number of patients with diabetes globally reach 537 million in 2021, and will increase to an estimated 783 million by 2045 (Sun et al., 2022).

Poorly controlled chronic hyperglycemia leads to various diabetic complications of the microvasculature and macrovasculature. Diabetic nephropathy (DN) is severe diabetic microvascular complication. Up to 30–40% of diabetic patients develop DN (Said et al., 2018). The main clinical characteristics are substantial proteinuria and a continuous decrease in glomerular filtration. An augmented accumulation of extracellular matrix (ECM) proteins also occurs (Xu et al., 2018). Eventually, nodular diabetic glomerulosclerosis is induced (Wang et al., 2018). DN has become the main risk factor for chronic kidney disease and end-stage renal disease (ESRD) (Sun et al., 2022) worldwide. Therefore, it is urgent to establish effective treatments to reduce its societal and financial burden.

The pathogenesis of DN is complicated and mainly includes changes in glomerular hypertrophy, mesangial matrix deposition, basement membrane thickening, tubulointerstitial fibrosis and atrophy. The mechanisms of DN are also complex, including the activation of oxidative stress (Baynes, 1991), reactive oxygen species (ROS) (Lapolla et al., 2005), transforming growth factor β1 (TGF-β1) (Aihara et al., 2010), inflammation (Luc et al., 2019), advanced glycation end products (AGEs) (Gross et al., 2020), apoptosis of renal tubular epithelial cells (Ding et al., 2021) and mitochondrial dysfunction (Xiao et al., 2017). However, the treatment of DN is facing challenges, such as drug side effects. Thus, natural product application may be a trend to cure DN. Traditional Chinese medicine (TCM) and healthy food have benefits in treating many diseases (Dai et al., 2020; Wu et al., 2020). In particular, TCMs usually have multiple active components that can affect multiple targets and pathways.

The main ingredient of Bailing Capsule is fermented *Cordyceps sinensis* (*C. sinensis*, syn. *Hirsutella sinensis*) powder. Bailing Capsule is approved by the State Food and Drug Administration of China (state medical license No. Z10910036). *C. sinensis* belongs to the family *Clavicipitaceae*, division *Ascomycotina*. It is a parasitic fungus. *C. sinensis,* which has multiple active constituents, including cordycepin and its derivatives, polysaccharides, trace elements, and mycelia. These chemical ingredients exert several physiological effects of resistance to oxidation, fibrosis, tumors, viruses, and inflammation. For centuries, *C. sinensis* has been widely used as a TCM. Recently, *C. sinensis* has been used in clinical practice to treat chronic kidney disease (CKD) (Deng, 2001). In animal models, *C. sinensis* reduced fasting blood glucose (Lu et al., 2019) and attenuated renal fibrosis (Du et al., 2015; Dong et al., 2019) and kidney function (Xiao et al., 2018; Lu et al., 2019).

Previous clinical research found that Bailing Capsule could reduce proteinuria and improve renal function in DN (Sheng et al., 2020), nephrotic syndrome (NS) (Xu et al., 2020), lupus nephritis patients (Ren et al., 2019) and renal transplant recipients (Wang et al., 2013). Moreover, Bailing Capsule combined with Western medicine, has better efficacy than Western medicine alone to treat kidney diseases by reducing urine protein and protecting kidney function (Hu et al., 2021; Yu et al., 2021). The mechanism research of Bailing Capsule on moderating kidney function is limited, including anti-inflammatory, anti-oxidative (Niu and Li, 2019), immune moderation (Ma and Yan, 2021).

Thus, the mechanism of the action of Bailing Capsule is still unclear and requires further investigation. Conventional pharmacological methods focused one kind or group of molecules. Whole transcriptome profiling of gene arrays is a useful tool to study complex pathways and drug targets (Logan et al., 2018) and has been widely used in the diabetes field (Zhang et al., 2019). It is important to reveal TCM in regulatory network and build a bridge between TCM and modern pharmacology at whole system level. Thus, we designed this study to determine the effect of Bailing Capsule on the kidney damage of DN rats induced by streptozotocin (STZ) and used a whole transcriptome gene array to identify the differentially expressed genes between the renal tissue of Bailing Capsule-treated group and DN group, screen the pathways and molecular targets, and explore the therapeutic mechanism of Bailing Capsule in delaying the progression of DN.

## Materials and methods

### Medicine

Bailing Capsule was used in our study. The composition of Bailing Capsule was fermented *C. sinensis* powder (Cs-C-Q80).

### Ultra-performance liquid chromatography analysis of bailing capsule

We performed the chromatographic separation of Bailing Capsule by using a Waters Acquity Ultra-Performance Liquid Chromatography (UPLC, Water Corp., Milford, MA) with a symmetrical C18 column (5 μm, 4.6 × 250 mm, Water Corp., Milford, MA). The chromatographic parameters were as follows: mobile phase A (0.1% phosphoric acid solution) and mobile phase B (methanol), flow rate of 1.0 ml/min, column temperature at 40°C, and detection wavelength of 260 nm with an injection volume of 5 μL. Gradient separation was based on the following: 0–5 min, 100% A, 10–15 min, 80% A, 15–16 min, 50% A.

### Animal treatments and diets

**FIGURE 1:**
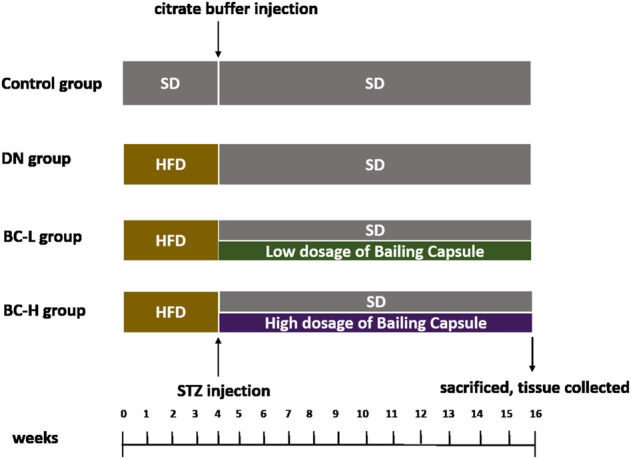
Schematic of group design. SD, standard diet; HFD, high-fat diet; DN, diabetic nephropathy; BC-L, low dosage of Bailing Capsule; BC-H, high dosage of Bailing Capsule.

Thirty-two male Sprague-Dawley (SD) rats, aged 5 weeks, were purchased from the Institute of Laboratory Animal Sciences, Chinese Academy of Medical Sciences and Peking Union Medical College, Beijing, China. All rats were housed in a specific pathogen-free facility at a temperature of 25 ± 1°C and a standard 12-h light/12-h dark cycle. Sterilized water and food was provided to the rats *ad libitum*. All of the animal procedures were approval by the Animal Care Committee of the Peking Union Medical Hospital Animal Ethics Committee (Project XHDW-2015-0051, 15 February 2015). DN rat model was developed by a high-fat diet (HFD) feeding combined with low dosage of streptozotocin (STZ) injection, according to previous studies (Danda et al., 2005; Liu et al., 2020; Guo et al., 2021). All rats were randomly divided into a normal control group (*n* = 8) fed a standard diet (SD, fat content, 10 kcal %, D12450B, Research Diets Inc., New Brunswick, NJ) and a DN model group (*n* = 24) fed a HFD (fat content, 60 kcal %, D12492, Research Diets Inc., New Brunswick, NJ), respectively. After 4 weeks, the DN model was induced by an intraperitoneal injection of 1% (g/100 ml) STZ (S0130, Sigma-Aldrich, St. Louis, MO) at a dose of 40 mg/kg in 0.1 mol/L citrate buffer, pH 4.5 (Furman, 2015; Nath et al., 2017; Liu et al., 2020). Fasting blood glucose (FBG) was assayed from the tail vein by an automatic glucometer (Contour TS, Bayer, Leverkusen, Germany) after 7 days. The rats with FBG >11.1 mmol/L were divided into the DN group (*n* = 8), the low dosage of Bailing Capsule (BC-L, *n* = 8) group, and the high dosage of Bailing Capsule (BC-H, *n* = 8) group. After disassembling the capsule shell of Bailing Capsule (Hangzhou Zhongmei East China Pharmaceutical Co., Hangzhou, China), the content was ground. The typical human daily dose of Bailing Capsule is 20 g per 60 kg of body weight. Thus, according to the formula d_rat_ = (37 × d_human_)/6, the corresponding dose of Bailing Capsule for rats is 2.06 g/kg per day. Therefore, the BC-L group and BC-H group were gavaged with Bailing Capsule at 1 and 2 g/kg/day (dissolved in saline), respectively. The gavage volume is 10 ml/kg body weight. The rats in the DN group and control group were given an equal volume of saline. At week 12 after treatment, all rats were sacrificed under anesthesia with an intraperitoneal injection of sodium pentobarbital (150 mg/kg). The kidneys were immediately collected. Figure 1 illustrates the study procedure.

### Measurement of body weight, kidney weight, and fasting blood glucose

At the end of the treatment, the body weight and kidney weight of the SD rats were recorded. Kidney hypertrophy was calculated as the ratio of the kidney weight to the body weight. After 6 h of fasting, FBG was determined using an automatic glucometer (Contour TS, Bayer, Leverkusen, Germany).

### Measurements of serum creatinine, blood urea nitrogen

After 6 h of fasting, blood was collected through the intraorbital retrobulbar plexus. Serum creatinine (OSR6178, Beckman Coulter Inc., Brea, CA), and BUN (OSR6234, Beckman Coulter Inc., Brea, CA) were measured by an automatic biochemical analyzer (AU5800, Beckman Coulter Inc., Brea, CA).

### Measurements of 24 h urinary protein and urinary albumin

The rats were housed in individual metabolic cages at the end of the 12-week treatment in order to collect 24 h urine samples. Urine protein (OSR6132, Beckman Coulter Inc., Brea, CA) and albumin (OSR6202, Beckman Coulter Inc., Brea, CA) levels were measured with a Beckman biochemical analyzer (AU5800, Beckman Coulter Inc., Brea, CA).

### Histological observation of the kidney

Kidney samples were fixed in 10% neutral-buffered formalin for 24 h and dehydrated with graded ethanol. The specimens were embedded in paraffin, cut into slices (5 μm thick), dewaxed, rehydrated. Then, hematoxylin and eosin (H&E), Periodic Acid Schiff (PAS) and Masson’s trichome stained samples were separately used for histopathological and collagen examination. The histological changes were observed using a digital microscope (Nikon, Tokyo, Japan). ImageJ 1.4 software (NIH, Bethesda, MA) was used for image analysis.

### Triglyceride assay in the kidney

According to Folch method (Folch et al., 1957), 100 mg kidney were ground with PBS and extracted with chloroform and methanol. After centrifugation, the contents of TG (120 μL) were determined at absorption wavelength 420 nm by spectrophotometry method (Solarbio, Beijing, China). The TG content was computed as following, TG concentration = Concentration_Standand_ × (Absorption Value_Test_−Absorption Value_Blank_) ÷ (Absorption Value_Standard_−Absorption Value_Blank_) ÷ Sample protein concentration.

### Oil Red O staining of the kidney

Oil Red O staining was used to detect lipid deposition in the rat renal tissue. Frozen kidney tissue sections (5 μm) were fixed in 10% formalin for 10 min, washed in distilled water and then stained in 1% Oil Red O solution (60% isopropanol) for 15 min. The sections were rinsed with 60% isopropanol for 2 min and counterstained with hematoxylin for 1 min. The stained sections were visualized using a digital microscope (Nikon, Tokyo, Japan), and the area of lipid deposition was assessed using ImageJ 1.4 software (NIH, Bethesda, MA) by converting the RGB image to an 8-bit grayscale image and adjusting threshold to analyze (Mehlem et al., 2013).

### RNA preparation and transcriptome microarray

Total RNA was collected from kidney tissues with an RNeasy RNA extraction kit (Qiagen, Valencia, CA). The RNA concentration, purity and integrity were determined by a Nanodrop™ ND-1000 and 1.0% agarose gel electrophoresis. Cy3-UTP-labeled cRNA was fragmented and hybridized to an Agilent Rat gene expression 4 × 44 K microarray (Agilent Technologies, Santa Clara, CA). The microarray was incubated for 17 h at 65°C in an Agilent Hybridization Oven. Then, the microarray was washed, fixed and scanned by using an Agilent DNA Microarray Scanner (G2505C, Agilent Technologies, Santa Clara, CA). Quantile normalization and subsequent data processing were performed using the GeneSpring GX v12.1 software package (Agilent Technologies, Santa Clara, CA).

### Data normalization and analysis

Differentially expressed genes with corrected *p* value <0.05 and fold change >1.5 were judged as statistically significant. The data obtained from the microarray have been deposited in the National Center for Biotechnology Information Gene Expression Omnibus (GEO) database (accession number GSE198021, https://www.ncbi.nlm.nih.gov/geo/query/acc.cgi?acc=GSE198021).

### Gene function enrichment analysis

Gene Ontology (GO) biological process and Kyoto Encyclopedia of Genes and Genomes (KEGG) pathway enrichment analyses were performed on the above differentially expressed genes by using Database for Annotation, Visualization and Integrated Discovery Software (Huang da et al., 2009) (DAVID, version 6.8, http://david.ncifcrf.gov/). Statistical differences of enriched GO and KEGG terms were defined as *p* < 0.05. The top 5 and 10 most significant GO and KEGG terms were selected and plotted, respectively.

### Quantitative real time polymerase chain reaction analysis

Total RNA and then cDNA were prepared from renal tissue using TRIzol reagent (Invitrogen, Carlsbad, CA) and reverse transcriptase (PrimeScipt™ RT reagent Kit, Takara Bio Inc., Shiga, Japan). *Acox1* (acyl-CoA oxidase 1), *Ppara* (peroxisome proliferator activated receptor alpha), *Scd* (stearoyl-CoA desaturase), and *Fasn* (fatty acid synthase) were analyzed by qPCR analysis using SYBR Green Mix Kit (Applied Biosystems, Foster City, CA) on the ABI ViiA 7 (Applied Biosystems, Carlsbad, CA). Relative changes in gene expression were calculated using the 2^-△△CT^ method. β-actin was used as an internal control. The primers used in this study are listed in Table 1.

**TABLE 1:** Primers used for qPCR.

Gene (rat)	Gene bank ID	Forward primer (5′ to 3′)	Reverse primer (3′ to 5′)	Product size (bp)
*Acox1*	NM_017340	TGT​CTG​TCA​CTT​CTG​TCG​CC	CGG​ACT​GCC​ATC​CAA​GAT​GT	135
*Ppara*	NM_013196	CTG​TCC​GCT​ACT​TCG​AGT​CC	TCA​AGG​GGA​CAA​CCA​GAG​GA	135
*Scd*	NM_139192	GTT​CTT​CAT​CGA​CTG​CAT​GGC	GAA​CAG​GAA​CTC​AGA​AGC​CCA	143
*Fasn*	NM_017332	GAG​CAC​TGA​TGA​GCA​CAC​CT	CCA​TCA​GGT​TTC​AGC​CCC​AT	111

*Acox1*, acyl-CoA oxidase 1; *Ppara*, peroxisome proliferator activated receptor alpha; *Scd*, stearoyl-CoA desaturase; *Fasn*, fatty acid synthase.

### Immunohistochemistry for ACOX1, PPARα, SCD, and FASN in the kidney

Immunohistochemistry staining was performed. Briefly, the sections were preincubated with 3% hydrogen peroxide and blocked with 2.5% normal horse serum. Then, the slides were incubated with anti-ACOX1 (ab184032, 1:200, Abcam, Cambridge, United Kingdom), PPARα (ab233078, 1:200, Abcam, Cambridge, United Kingdom), SCD (ab19862, 1:300, Abcam, Cambridge, United Kingdom), and FASN (ab22759, 1:200, Abcam, Cambridge, United Kingdom) primary antibodies overnight at 4°C and then with the secondary antibody (GB25301, 1:500, Servicebio Co., Ltd., Wuhan, China) at room temperature for 60 min. ImageJ 1.4 software (NIH, Bethesda, MA) was used to quantify the protein expression.

### Western blot analysis of ACOX1, PPARα, SCD, and FASN in the kidney

Protein was extracted from renal tissues. Membranes were incubated with primary antibodies against ACOX1 (ab184032,1:1000, Abcam, Cambridge, United Kingdom), PPARα (ab126285, 1:1000, Abcam, Cambridge, United Kingdom), SCD (ab39969, 1:1000, Abcam, Cambridge, United Kingdom), FASN (ab128870, 1:10000, Abcam, Cambridge, United Kingdom), and β-actin (ab8226, 1:1000, Abcam, Cambridge, United Kingdom) at 4°C overnight, and then the membranes were further incubated with horseradish peroxidase-conjugated secondary antibodies (GB25301, 1:5000, Servicebio Co., Ltd., Wuhan, China). Protein band densities were detected by chemiluminescence (Cell Signaling Technology, Danvers, MA), followed by scanning using an Epson V300 scanning system (Epson, Suwa, Japan), analyzed by AlphaEaseFC software (Alpha Innotech, San Leandro, CA), and quantified by ImageJ 1.4 software (NIH, Bethesda, MA).

### Statistical analysis

The results are shown as the mean ± SD. Comparisons between groups were assessed using one-way ANOVA followed by Tukey’s post hoc test (GraphPad Prism, v 7.0, GraphPad Software, Inc., La Jolla, CA), and the significance level was set at *p* values <0.05.

## Results

### Ultra-performance liquid chromatography of bailing capsule

**FIGURE 2:**
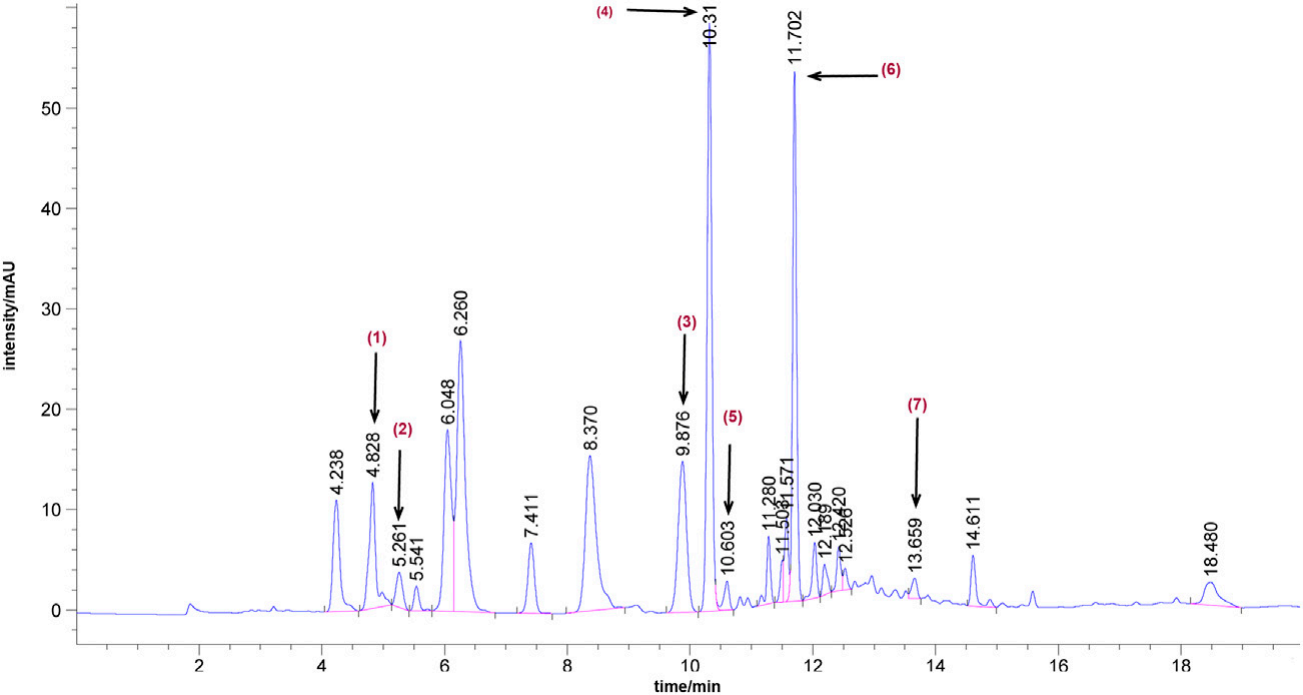
Representative UPLC chromatograms of Bailing Capsule. 1) adenine (390.85 μg/ml), 2) uracil (73.41 μg/ml), 3) uridine (1342.18 μg/ml), 4) adenosine (2136.96 μg/ml), 5) 2′-deoxyadenosine (203.36 μg/ml), 6) guanosine (20.25 μg/ml), and 7) thymidine (1.46 μg/ml).

Seven main components in Bailing Capsule were confirmed by UPLC analysis, including 1) adenine (390.85 μg/ml), 2) uracil (73.41 μg/ml), 3) uridine (1342.18 μg/ml), 4) adenosine (2136.96 μg/ml), 5) 2′-deoxyadenosine (203.36 μg/ml), 6) guanosine (20.25 μg/ml), and 7) thymidine (1.46 μg/ml, Figure 2).

### Bailing capsule ameliorates fasting blood glucose in diabetic nephropathy rats

**FIGURE 3:**
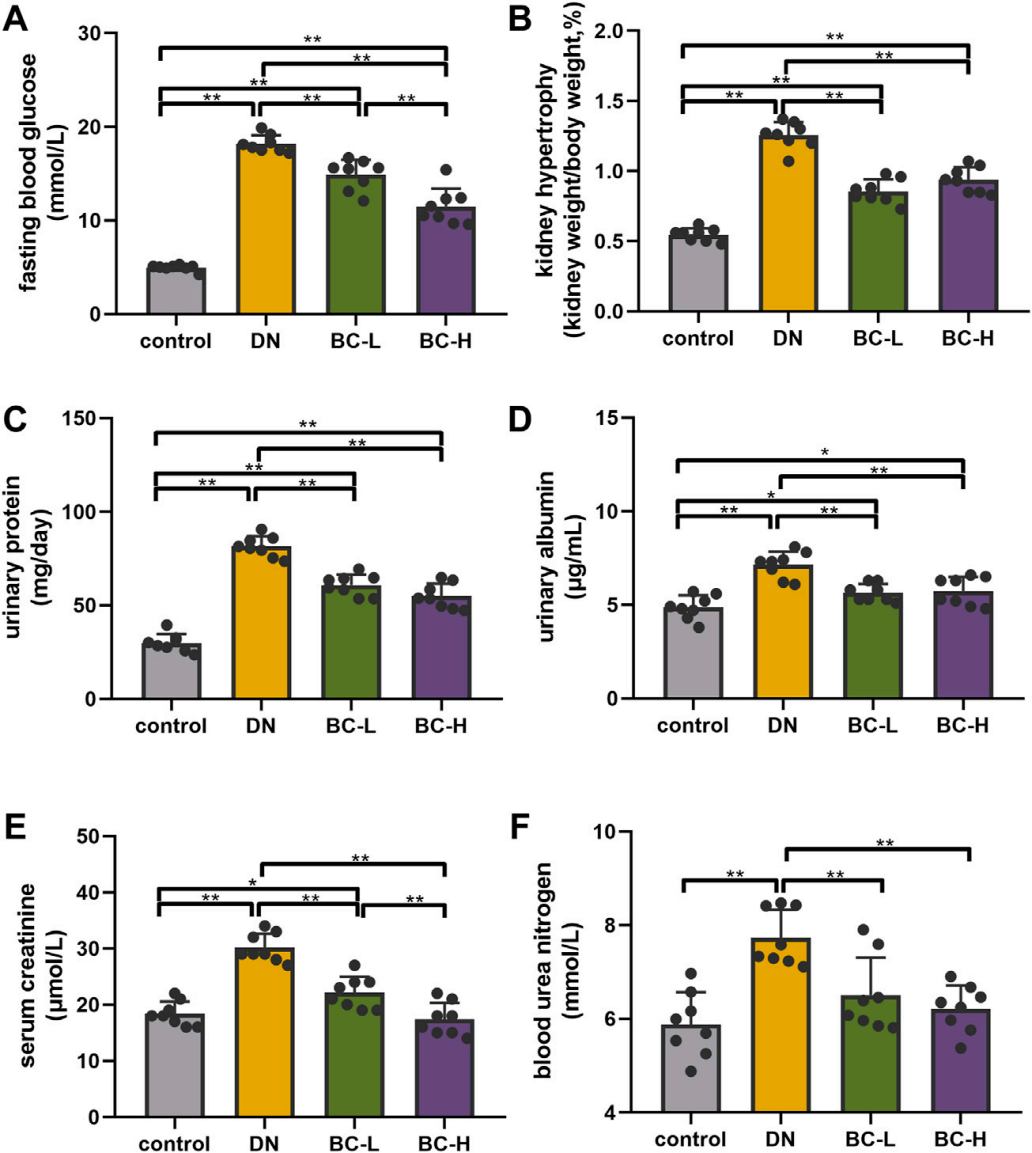
Bailing Capsule improved blood glucose and kidney function. **(A)** Bailing Capsule significantly reduced fasting blood glucose (FBG). **(B)** Bailing Capsule significantly reduced kidney hypertrophy. **(C)** Bailing Capsule significantly reduced 24 h urinary protein. **(D)** Bailing Capsule significantly reduced urinary albuminuria. **(E)** Bailing Capsule significantly reduced serum creatinine. **(F)** Bailing Capsule significantly reduced blood urea nitrogen (BUN). DN, diabetic nephropathy; BC-L, low dosage of Bailing Capsule; BC-H, high dosage of Bailing Capsule. Data are expressed as mean ± SD (*n* = 8). *p < 0.05 and **p < 0.01.

FBG in the DN groups was remarkably elevated compared with the control group, while Bailing Capsule treatment significantly reduced FBG (*p* < 0.01, Figure 3A).

### Bailing capsule improved kidney hypertrophy and renal function in diabetic nephropathy rats

Kidney hypertrophy is expressed as a ratio of kidney weight to body weight. DN rats showed a significant increase in kidney hypertrophy compared with the control group (*p* < 0.01, Figure 3B), while the administration of Bailing Capsule to DN rats significantly reduced kidney hypertrophy (*p* < 0.01, Figure 3B). As shown in Figures 3C–F, DN rats exhibited elevated 24 h urinary protein, urinary albuminuria, serum creatinine, and BUN, compared to control rats (*p* < 0.01, Figures 3C–F). Bailing Capsule treatment significantly reduced 24 h urinary protein, urinary albuminuria, serum creatinine, and BUN (*p* < 0.01, Figures 3C–F).

### Bailing capsule moderates histopathological changes in diabetic nephropathy rats

**FIGURE 4:**
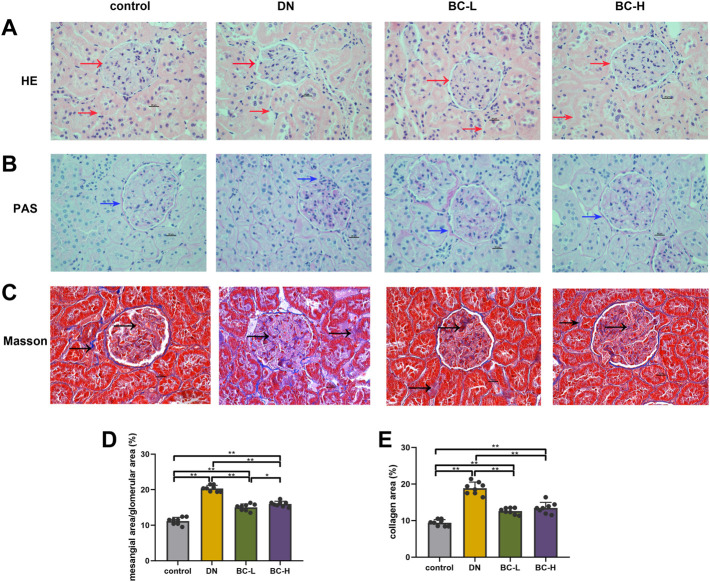
Bailing Capsule alleviated morphological changes of kidney. **(A)** H&E staining (400 X) of kidney showed morphological changes in DN rat kidney, including glomerular basement membrane thickening and tubular basement membrane thickening. Bailing Capsule treatment alleviated these changes. Red arrow showed the basement membrane. **(B)** PAS staining (400 X) of kidney showed morphological changes in DN rat kidney, including glomerular mesangial matrix accumulation. Bailing Capsule treatment alleviated these changes. Blue arrow showed glomerular mesangial matrix accumulation. **(C)** Masson staining (400 X) of kidney showed collagen fiber deposition. Bailing Capsule relieved renal fibrosis in DN rats. The blue stain by the black arrow indicated is collagens fiber. Scale bar = 20 μm. **(D)** The quantification of mesangial area/glomerular area (%) of kidney. **(E)** The quantification of collagen area (%) of kidney. DN, diabetic nephropathy; BC-L, low dosage of Bailing Capsule; BC-H, high dosage of Bailing Capsule. Data are expressed as mean ± SD (*n* = 8). *p < 0.05 and **p < 0.01.

In Figures 4A,B,D, H&E and PAS staining show significant morphological changes in DN rat kidney, including glomerular hypertrophy, glomerular mesangial matrix accumulation, glomerular basement membrane thickening and tubular basement membrane thickening (*p* < 0.01). Bailing Capsule treatment alleviated these changes (*p* < 0.01, Figures 4A,B,D). Masson staining showed that glomerular and tubulointerstitial fibrosis occurred in DN rat kidney (*p* < 0.01, Figures 4C,E). However, the degree of fibrosis was relieved after administration of Bailing Capsule (*p* < 0.01, Figures 4C,E).

### Bailing capsule administration reduces kidney lipid accumulation in diabetic nephropathy rats

**FIGURE 5:**
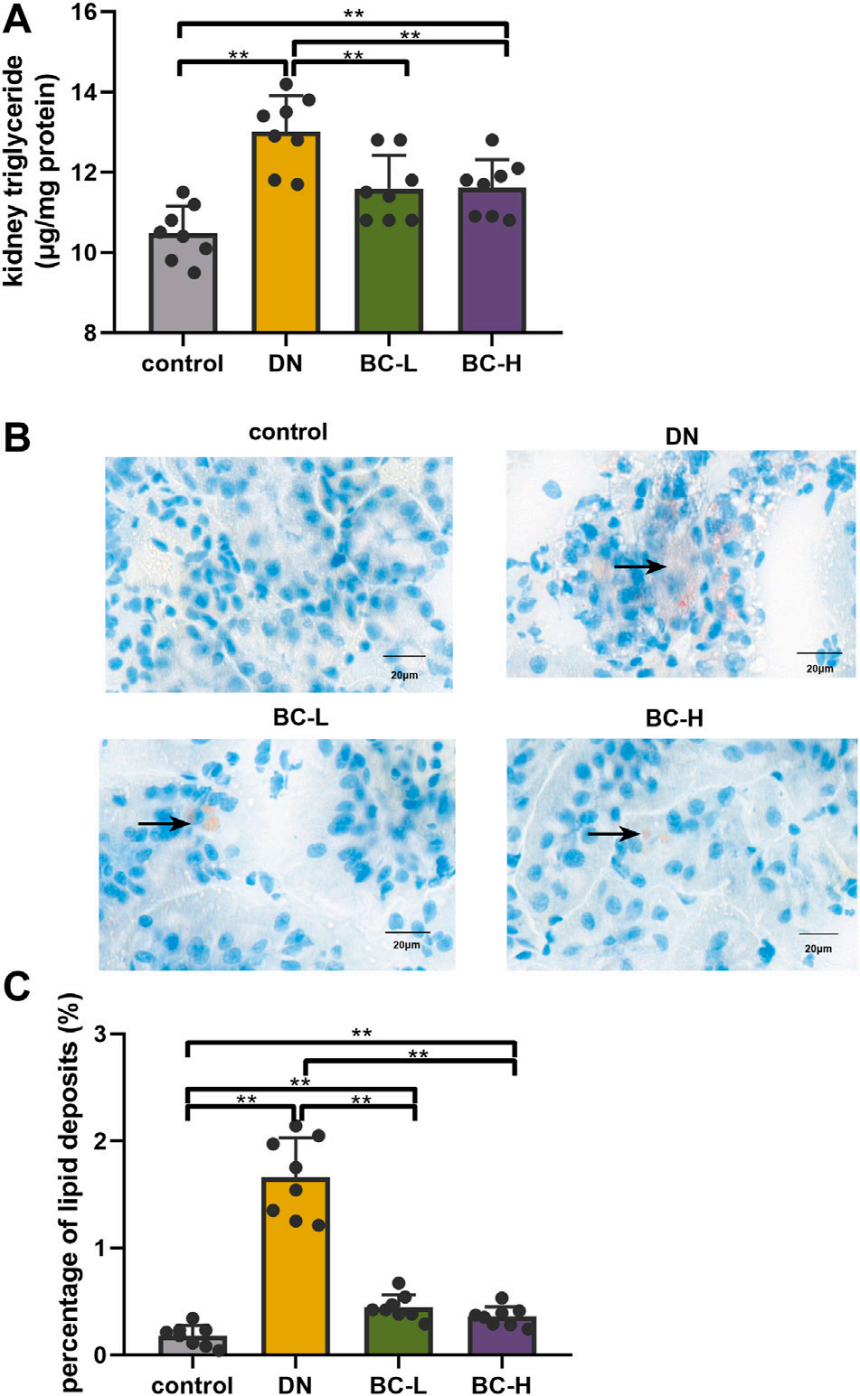
Bailing Capsule reduced kidney lipid accumulation. **(A)** Kidney triglyceride (TG) content. Bailing Capsule reduced kidney TG contents in DN rats. **(B)** Lipid droplets were detected by Oil Red O staining (400 X). Bailing Capsule reduced the percentage of lipid deposits in DN rats. Black arrow showed the lipid droplets. Scale bar = 20 μm. **(C)** The quantification of percentage of lipid deposits. DN, diabetic nephropathy; BC-L, low dosage of Bailing Capsule; BC-H, high dosage of Bailing Capsule. Data are expressed as mean ± SD (*n* = 8). **p < 0.01.

Kidney TG contents were increased significantly in DN rats compared with those in the control group (*p* < 0.01, Figure 5A), and this change was blocked in Bailing Capsule-treated groups (*p* < 0.01, Figure 5A). Moreover, enhanced lipid deposition was observed in DN kidneys, compared to control kidneys, as assessed by Oil Red O staining (Figures 5B,C). Bailing Capsule reduced the percentage of lipid deposits in the kidney of diabetic rats (*p* < 0.01, Figures 5B,C).

### Renal transcriptome analysis

To clarify the underlying mechanisms of Bailing Capsule on renal protection, we applied transcriptomic analysis to compare the kidneys of the BC-H group and DN group (*n* = 3). In this study, a total of 946 differentially expressed genes (DEGs) were identified in the BC-H group versus the DN group, 498 of which were upregulated and 448 of which were downregulated (fold change >1.5, *p* < 0.05).

### Gene function enrichment analysis

**FIGURE 6:**
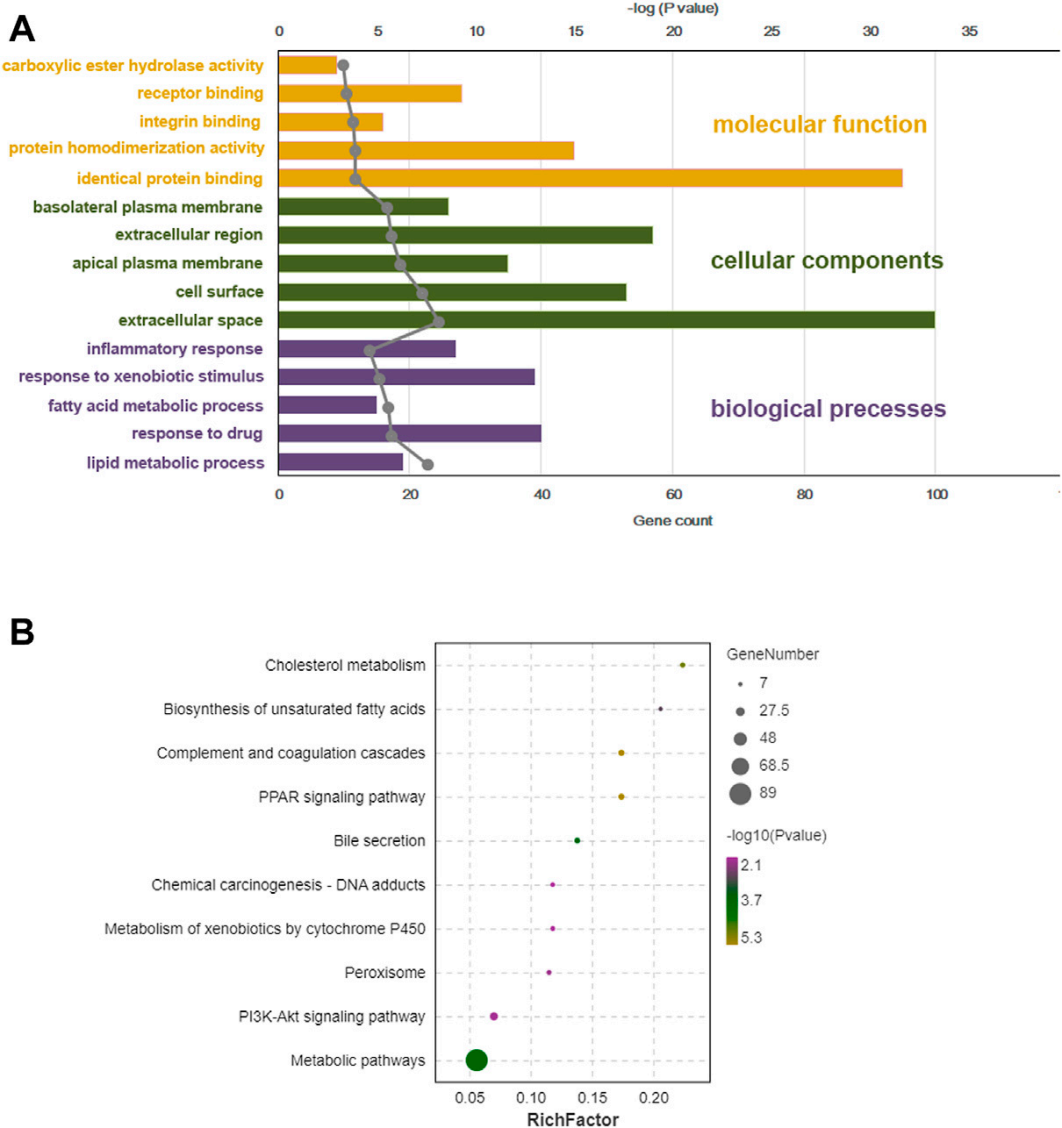
Bioinformation analysis of enriched GO (gene ontology) term and KEGG (Kyoto Encyclopedia of Genes and Genomes) pathways of differentially expressed genes from BC-H (high dosage of Bailing Capsule) group vs. DN (diabetic nephropathy) group. **(A)** Top 5 significant GO term of DEGs (differentially expressed genes) identified by transcriptional analysis in each catalogue. **(B)** KEGG enrichment analysis for DEGs identified by transcriptomics analysis.

**FIGURE 7:**
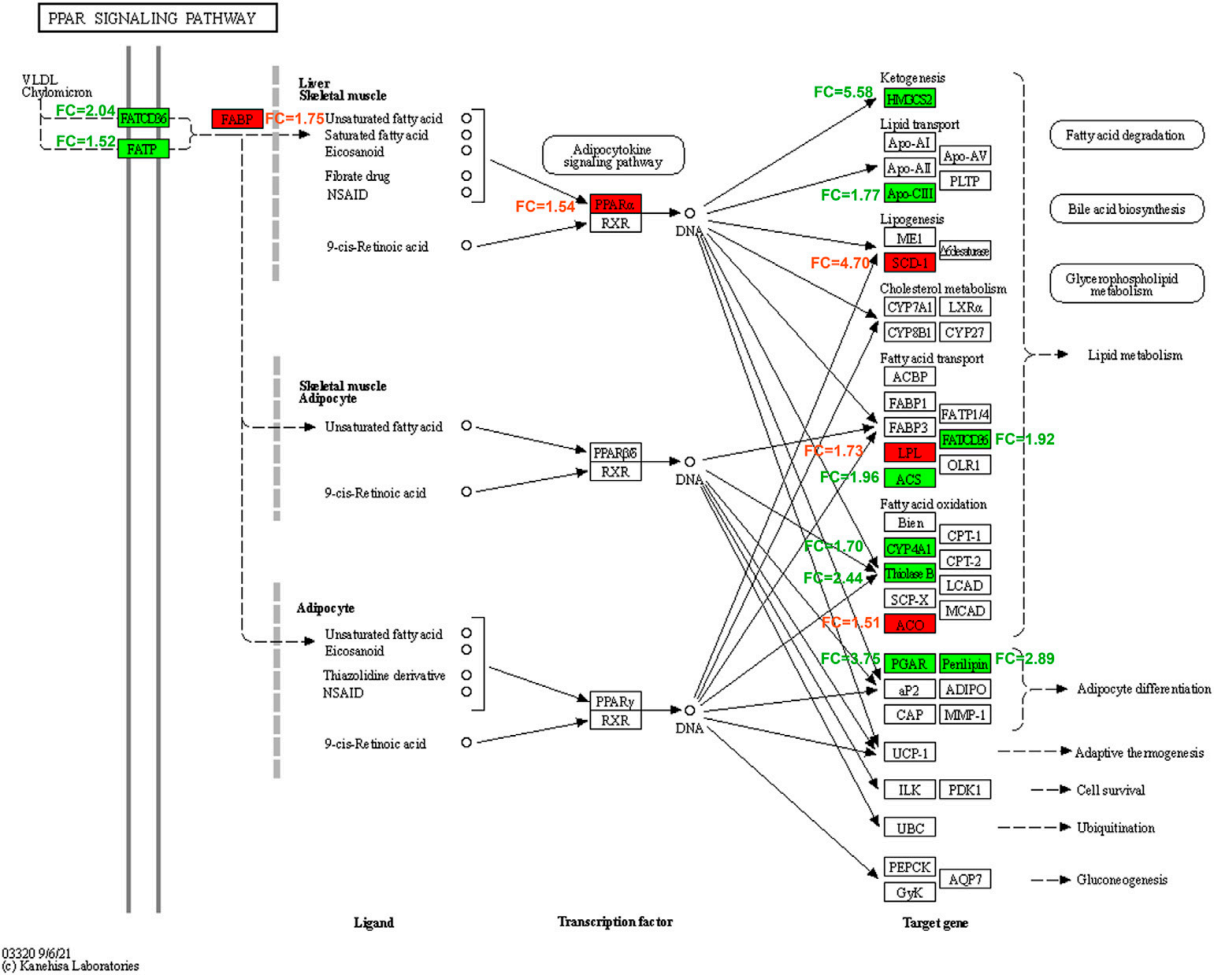
PPAR (peroxisome proliferator activated receptor) signaling pathway affected by Bailing Capsule in DN (diabetic nephropathy) rats. All the different expression genes of BC-H (high dosage of Bailing Capsule) group vs. DN group in PPAR pathway. Red represents up-regulated; green represents down-regulated, white represents no significant change. FC, fold change in BC-H vs. DN group.

GO function and KEGG pathway enrichment analyses of differentially expressed genes were performed. The top 5 enriched GO biological processes in each catalog included lipid metabolic process, response to drug, fatty acid metabolic process, response to xenobiotic stimulus, inflammatory response in biological processes, extracellular space, cell surface, apical plasma membrane, extracellular region, basolateral plasma membrane in cellular components, identical protein binding, protein homodimerization activity, integrin binding, receptor binding, and carboxylic ester hydrolase activity in molecular function (*p* < 0.001, Table 2, Figure 6A). The top 10 enriched KEGG pathways included complement and coagulation cascades, PPAR signaling pathway, cholesterol metabolism, metabolic pathways, bile secretion, biosynthesis of unsaturated fatty acids, peroxisome, phosphoinositide 3-kinase (PI3K)-Akt signaling pathway, chemical carcinogenesis, and metabolism of xenobiotics by cytochrome P450 (*p* < 0.01, Table 3, Figure 6B). All DEGs in the PPAR pathway are shown in Figure 7.

**TABLE 2:** Top 5 enriched GO terms in each catalog associated with differentially expressed genes in Bailing capsule-treated group vs. DN group (*p* < 0.001).

Term ID	Term name	Count	*p* value	Involved genes	Fold enrichment	FDR	Catalog
GO:0006629	lipid metabolic process	19	2.42 × 10^−8^	*Slc16a1, Ptprn2, Acsl1, Mogat2, Plcl1, Mttp, Pon1, Cidea, Trpv1, Fabp5, Aadac, Acox1, Apoc2, Dpep2, Hacl1, Apoe, Angptl4, Apob, Enpp6*	5.16	8.14 × 10^−5^	biological processes
GO:0042493	response to drug	40	1.72 × 10^−6^	*Alas2, Cdkn1a, Mgst1, Lpl, Wfdc1, Ptprm, Pdgfa, Slco1a6, Hsd11b2, Mat2a, Oxct1, Erbb4, Cd36, Hmgcs2, Abcb1a, Abcb1b, Tgif1, Ntrk1, Abcc2, Cox8b, Gstm1, Acsl1, Itga3, Mgmt, Apoc3, Mthfr, Dusp6, Gnao1, Hadha, Sfrp2, Ccne1, Gsta3, Apoc2, Acot2, Cyp1a1, Txnip, Pemt, Plin2, Cftr, Tp73*	2.32	0.0027	biological processes
GO:0006631	fatty acid metabolic process	15	2.49 × 10^−6^	*Acbd7, Faah, Acot7, Acsl1, Acot12, Lpl, Baat, Acadsb, Cyp1a1, Acot2, Hacl1, Cd36, Gpat2, Ppara, Acot4*	4.81	0.0027	biological processes
GO:0009410	response to xenobiotic stimulus	39	6.99 × 10^−6^	*Alas2, Cdkn1a, Mgst1, Lpl, Wfdc1, Ptprm, Pdgfa, Slco1a6, Hsd11b2, Mat2a, Oxct1, Erbb4, Cd36, Hmgcs2, Abcb1a, Abcb1b, Tgif1, Ntrk1, Abcc2, Cox8b, Gstm1, Acsl1, Itga3, Mgmt, Apoc3, Mthfr, Dusp6, Gnao1, Hadha, Sfrp2, Ccne1, Apoc2, Acot2, Cyp1a1, Txnip, Pemt, Plin2, Cftr, Tp73*	2.22	0.0058	biological processes
GO:0006954	inflammatory response	27	2.17 × 10^−5^	*Pxk, Calca, Serpina1, Sgms1, Fpr2, Cxcl2, Kng1, C4a, Hyal1, Hrh4, Pdpn, Abcc2, Igfbp4, Rarres2, Apoc3, Trpv1, Il17re, Tirap, Cxcl11, Vnn1, Fasn, Prkd1, Bmpr1b, Klkb1, S100a8, Epha2, Tp73*	2.56	0.014	biological processes
GO:0005615	extracellular space	100	7.11 × 10^−9^	*Tfrc, Cyp4a1, Clu, C4a, Efemp2, Efemp1, Tnn, Igfbp1, Cpa1, Igfbp5, Igfbp4, Sema6d, Fibcd1, Defb11, Bcan, Rbp4, Sfrp2, Col4a1, S100a9, S100a8, Calca, Pdgfa, Lpl, Kng1, Klk7, Loc290595, Apoe, Apob, Cga, Fgf21, Ambp, Lrrn2, Rarres2, Inhbb, Igf1, Cxcl11, Bmp1, Tg, Artn, Scgb3a2, Col9a1, Lrrn1, Lgals3bp, Pigr, Serpina1, Wnt2b, Cpxm2, Wfdc1, Cpz, Cxcl2, Areg, Serpina4, Ces2c, Hyal1, Tctn1, Emilin1, Mbl1, Fgb, Fga, Myoc, Vwf, Nrg1, Apoa4, Rt1-Ce5, Nrg2, F2, F3, Serpinb5, Grem2, Ceacam1, Tff3, Angptl4, Pon3, Pon1, Sema3b, Tfpi, Defb42, Cpn1, Il1rl1, Wnt11, Slit3, Slit2, Gc, S100g, Angpt1, Gdf15, Apoc3, Colq, Apln, Kitlg, Epgn, Fabp5, Ces1d, Cfhr2, Ces1e, Apoc2, Ghrl, Ptx3, Ccl28, Klkb1*	1.80	3.52 × 10^−6^	cellular components
GO:0009986	cell surface	53	4.98 × 10^−8^	*Tfrc, Itgb2, Pkd2l1, Slc4a1, Slc2a4, Rt1-Db1, Clu, Areg, Grm7, Tnn, Itgb8, Itgb7, Cd36, Scn5a, Kcnh1, Kcnh2, Fgb, Fga, Gpr37, Entpd2, Abcc2, Fcer1g, Rgd1565355, Itga3, Sema6d, Apoa4, Rt1-Ce5, F3, Bcan, Vcan, Ceacam1, Tnmd, Rt1-Bb, Adtrp, Cryab, Cftr, Epha2, Kcne1, Pdgfa, Lpl, Tfpi, Il1rl1, Slamf9, Gpc3, Mbp, Kcnn2, Apoe, Slit2, Abcb1a, Ntrk1, Ambp, Bst2, Fgfr2*	2.28	1.23 × 10^−5^	cellular components
GO:0016324	apical plasma membrane	35	6.01 × 10^−7^	*Slc26a2, Kcne1, Slc22a2, Slc2a2, Atp12a, Slc7a1, Slc4a5, Slc9a3r2, Gnat3, Pdpn, Ceacam20, Cd36, Abcb1a, Abcb1b, Mpdz, Slc39a4, S100g, Slc16a1, Abcc2, Rgd1565355, Anxa13, Lhfpl5, Nrg1, Pard3b, Bst2, Atp4b, Slc6a6, Ceacam1, Trpv6, Cdhr2, Slc26a4, Rhcg, Atp6v0d2, Slc26a6, Cftr*	2.62	9.91 × 10^−5^	cellular components
GO:0005576	extracellular region	57	1.74 × 10^−6^	*Apol9a, Tfrc, Clu, C4a, Esm1, Smpd5, Eva1c, Enpp6, Adamts6, Tgm2, Ccdc39, Vwf, Igfbp5, Il1r2, Nrg1, Nrg2, Bcan, Vcan, Ccdc80, Sfrp2, Col4a1, Tff3, Angptl4, Cryab, Zp4, Calca, Dpt, Itih1, Tfpi, Ltbp1, Kng1, Coch, Loc290595, Wnt11, Spock2, Lcn10, Apoe, Slit2, Cga, Gstm1, Gdf15, Rarres2, Gzmb, Apoc3, Isg15, Inhbb, Apln, Efna1, Igsf10, Kitlg, Nmu, Scgb3a2, Ghrl, Fgf13, Ccl28, Scrg1, Klkb1*	1.97	2.16 × 10^−4^	cellular components
GO:0016323	basolateral plasma membrane	26	2.91 × 10^−6^	*Tfrc, Slc22a2, Mttp, Slc2a2, Slc4a1, Atp12a, Aqp3, Slc7a1, Slc4a5, Nkd2, Erbb4, Pdpn, S100g, Slc16a1, Slc10a1, Itga3, Anxa13, Slc16a12, Cask, Ank2, Slc6a6, Lrrc7, Slco1a1, Rhcg, Slc26a6, Cftr*	2.95	2.88 × 10^−4^	cellular components
GO:0042802	identical protein binding	95	1.20 × 10^−4^	*Clic6, Faah, Serpina1, Tfrc, Fmr1, Lect2, Ptprm, Pkd2l1, Grik2, Dnph1, Aqp3, Fgfrl1, Syne1, Gls, Grm7, Creb3l3, Tnn, Tnfaip8l1, Emilin1, Hmgcs2, Sult4a1, Mbl1, Tgm2, Kcnh1, Kcnh2, Acot7, Entpd2, Fcer1g, Vwf, Acot12, Apoa4, Mat1a, Bhlha15, Sdcbp2, Tirap, Grem2, Ceacam1, Prpf3, Padi3, Tff3, Hprt1, Prkd1, Angptl4, Rhcg, Cryab, Dctpp1, Fbp2, Zp4, Prps2, Calca, Mgst1, Pdgfa, Fhit, Cryz, Sav1, Slc9a3r2, Mat2a, Oxct1, Prdx1, Hacl1, Apoe, Slit2, Gbp2, Skil, Cap2, Atp6v1c2, Cd276, E2f8, Ntrk1, Gstm2, Slc16a1, Gstm1, Angpt1, Plk1, Mx1, Trpv1, Acadsb, Esr1, Tm6sf2, Apln, Ski, Dazl, Bst2, Bmp1, Tg, Fabp5, Trpv6, Sp1, Apoc2, Fasn, Ptx3, Mcu, Fgfr2, Slc26a6, Tp73*	1.46	0.053	molecular function
GO:0042803	protein homodimerization activity	45	1.25 × 10^−4^	*Prps2, Pon3, Tfrc, Fmr1, Pon1, Lpl, Cib2, Pdgfa, Pdxp, Slc4a1, Dnph1, Syne1, Add2, Efemp2, Creb3l3, Oxct1, Erbb4, Kcnn2, Apoe, Slit2, Ect2, Gbp2, Pdlim4, Mbl1, Kcnh2, Ntrk1, Gstm2, Cadm3, Acot7, Gstm1, Ambp, Gdf15, Glce, Cidea, Inhbb, Apoa4, Sdcbp2, Bst2, Tpst2, Ceacam1, Adrb3, Sp1, Acox1, Cryab, Fgfr2*	1.83	0.053	molecular function
GO:0005178	integrin binding	16	1.60 × 10^−4^	*Egfl6, Syk, Vwf, Itga3, Itgb2, Cib2, Isg15, Nrg1, Igf1, Esm1, Sfrp2, Gpnmb, Tnn, Lgals12, Itgb8, Itgb7*	3.17	0.053	molecular function
GO:0005102	receptor binding	28	3.47 × 10^−4^	*Calca, Lpl, Clu, Kng1, Syne1, Slc9a3r2, Pdpn, Itgb8, Nefm, Apoe, Cd276, Fgb, Fga, Gstm2, Srms, Syk, Angpt1, Rarres2, Fibcd1, Cask, Nrg1, Rt1-Ce5, Nrg2, F2, Gnao1, Tg, Artn, Angptl4*	2.12	0.0807	molecular function
GO:0052689	carboxylic ester hydrolase activity	9	4.90 × 10^−4^	*Ces2c, Acot8, Nceh1, Acot7, Ces1d, Ces1e, Acot2, Acot12, Baat*	4.82	0.080	molecular function

**TABLE 3:** Top 10 enriched KEGG pathways associated with differentially expressed genes in Bailing Capsule-treated group vs. DN group (*p* < 0.01).

Pathway ID	Pathway term	Count	*p* value	Involved genes	Fold enrichment	FDR
rno04610	Complement and coagulation cascades	15	4.62 × 10^−6^	*Fgb, Fga, Serpina1, Vwf, Itgb2, F2, Tfpi, F3, Clu, Kng1, C4a, Cfhr2, Klkb1, Mbl1, Loc100361907*	4.5	6.84 × 10^−4^
rno03320	PPAR signaling pathway	15	4.62 × 10^−6^	*Acsl1, Rgd1565355, Apoc3, Lpl, Cyp4a1, Acaa1b, Fabp5, Scd, Acox1, Plin2, Cd36, Hmgcs2, Angptl4, Ppara, Slc27a2*	4.5	6.84 × 10^−4^
rno04979	Cholesterol metabolism	11	1.40 × 10^−5^	*Mylip, Nceh1, Rgd1565355, Apoc2, Lpl, Apoc3, Apoa4, Apoe, Cd36, Angptl4, Apob*	5.79	0.0013
rno01100	Metabolic pathways	89	1.87 × 10^−4^	*Dgkg, Alas2, Gda, Mogat2, Pik3c2g, Cyp4a1, Hnmt, Atp12a, Eno4, Ftcd, Tm7sf2, Gls, Hyal1, Car15, Lrat, Hmgcs2, Enpp6, Lbr, Acot8, Cox8b, Entpd2, Acsl1, Glce, Lancl1, Amt, Acot12, Pipox, Ugt2b17, Mat1a, Baat, Sirt3, Hadha, Acox1, Itpka, Cyp2a1, Rgn, Acot2, Loc688321, Blvrb, Hprt1, L2hgdh, Atp6v0d2, St6galnac4, Dctpp1, Fbp2, Acot4, Blvra, Prps2, Sgms1, Pde1a, Mgst1, Ak2, Pdxp, Acaa1b, Fhit, Papss2, Hsd11b2, Neu3, Cyp3a9, Aanat, Mat2a, Cyp2c11, Oxct1, Aldh3b1, Hsd17b2, Rgd1559459, Hyi, Gpat2, B4galnt3, Atp6v1g3, Atp6v1c2, Cyp2j4, Gstm2, Gstm1, Mthfr, Acadsb, Sgpp2, Atp4b, Bhmt, Cyp24a1, Vnn1, Ggcx, Gsta3, Scd, Fasn, Cyp1a1, Pemt, Dhcr7, Galk1*	1.43	0.0138
rno04976	Bile secretion	13	2.52 × 10^−4^	*Abcc2, Slc10a1, Ugt2b17, Slco1a6, Baat, Slc4a5, Nceh1, Slco1a1, Rgd1559459, Kcnn2, Abcb1a, Abcb1b, Cftr*	3.56	0.014
rno01040	Biosynthesis of unsaturated fatty acids	7	0.0017	*Acot7, Scd, Acox1, Acot2, Acaa1b, Baat, Acot4*	5.31	0.084
rno04146	Peroxisome	10	0.0062	*Acot8, Pex19, Acsl1, Acox1, Prdx1, Pipox, Hacl1, Acaa1b, Baat, Slc27a2*	2.96	0.259
rno04151	PI3K-Akt signaling pathway	24	0.0075	*Ntrk1, Cdkn1a, Syk, Angpt1, Vwf, Itga3, Pdgfa, Igf1, Efna5, Areg, Efna1, Kitlg, Creb3l3, Ccne1, Tnn, Erbb4, Col4a1, Col9a1, Itgb8, Itgb7, Jak3, Fgfr2, Fgf21, Epha2*	1.79	0.259
rno05204	Chemical carcinogenesis - DNA adducts	9	0.0087	*Cyp3a9, Gstm2, Gstm1, Cyp2c11, Gsta3, Cyp1a1, Mgst1, Rgd1559459, Ugt2b17*	3.05	0.259
rno00980	Metabolism of xenobiotics by cytochrome P450	9	0.0087	*Gstm2, Gstm1, Gsta3, Aldh3b1, Cyp1a1, Loc688321, Mgst1, Rgd1559459, Ugt2b17*	3.05	0.259

### Real-time PCR validation of transcriptome analysis

**FIGURE 8:**
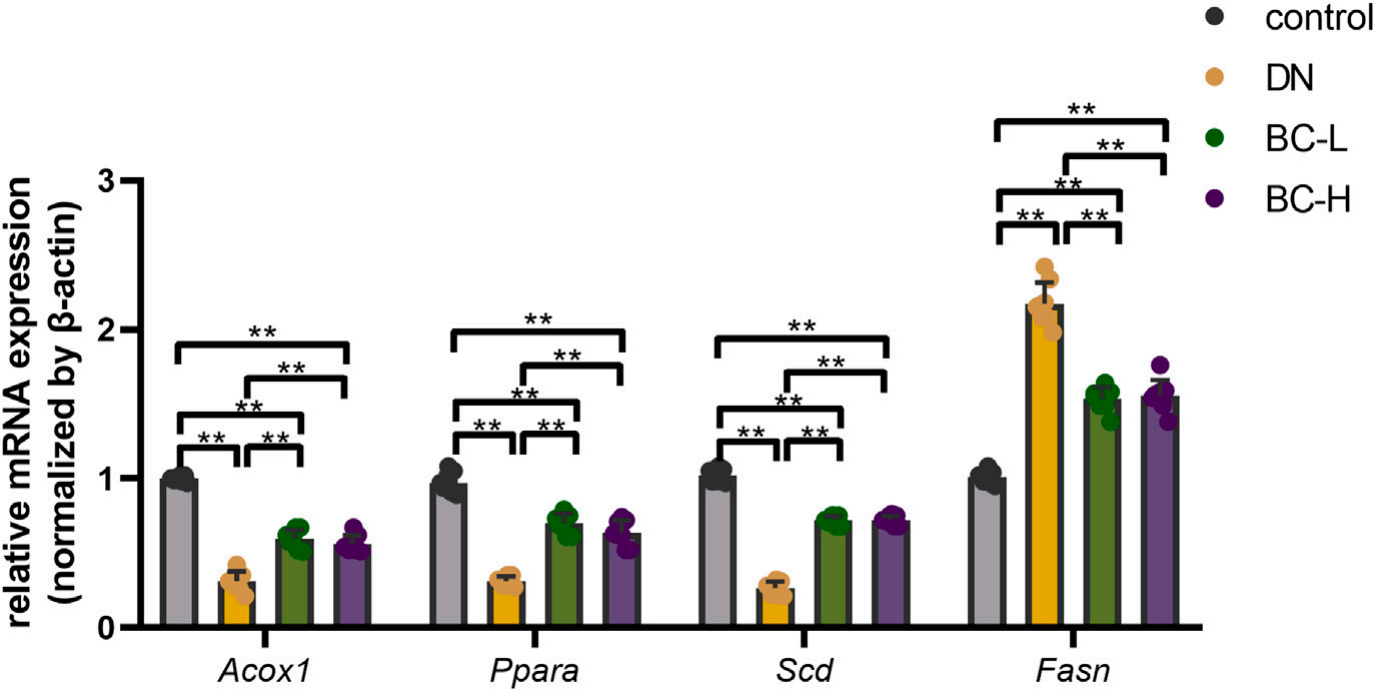
Real-time PCR analysis for main DEGs (differentially expressed genes) expression. Bailing Capsule increased the mRNA levels of *Acox1* (acyl-CoA oxidase 1), *Ppara* (peroxisome proliferator activated receptor α), *Scd* (stearoyl-CoA desaturase) and inhibited the mRNA levels of *Fasn* (fatty acid synthase) in DN (diabetic nephropathy) rats. DN, diabetic nephropathy; BC-L, low dosage of Bailing Capsule; BC-H, high dosage of Bailing Capsule. Data are expressed as mean ± SD (*n* = 8). **p < 0.01.

Because the PPAR signaling pathway was among the top-ranked KEGG pathways associated with differentially expressed genes in the BC-H group vs. the DN group, we selected *Acox1*, *Ppara*, *Scd*, and *Fasn* genes in the PPAR pathway to compare their expression among the four different groups using real-time PCR. As shown in Figure 8, the mRNA levels of typical PPAR pathway genes, such as *Acox1*, *Ppara*, *Scd*, and *Fasn*, were determined by RT-qPCR. In DN rats, the mRNA levels of *Acox1*, *Ppara*, and *Scd* were significantly reduced while *Fasn* was obviously elevated compared to the control group, and Bailing Capsule treatment moderated the DN-induced changes in their transcript levels (*p* < 0.01, Figure 8). The results of the real-time PCR experiments for these genes were consistent with the transcriptome profiling.

### Effects of bailing capsule on the peroxisome proliferator activated receptor pathway in diabetic nephropathy rats determined by immunohistochemistry staining

**FIGURE 9:**
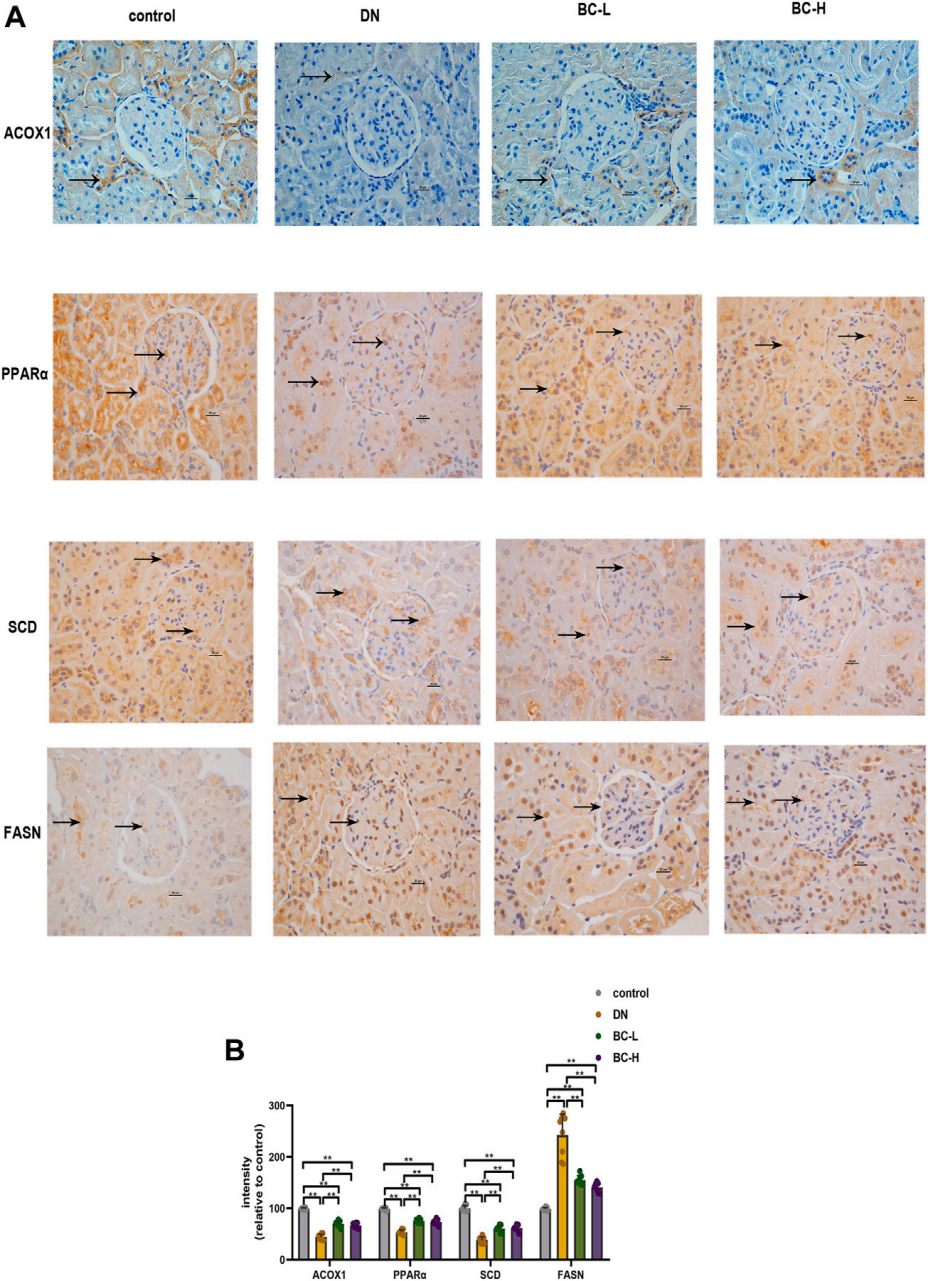
Effect of Bailing Capsule treatment on the expression levels of PPAR (peroxisome proliferator activated receptor) pathway in kidneys of rats shown by immunohistochemistry staining. **(A)** Representative immunohistochemistry staining for ACOX1, PPARα, SCD, and FASN (400 X). Scale bar = 20 μm. Black arrows showed ACOX1 (+), PPARα (+), SCD (+) and FASN (+) cells in the kidney. Immunohistochemistry staining showed that Bailing Capsule increased the protein levels of ACOX1, PPARα, SCD and inhibited the protein levels of FASN in DN rats. **(B)** Intensity of ACOX1 (+), PPARα (+), SCD (+) and FASN (+) cells in kidney of rats. DN, diabetic nephropathy; BC-L, low dosage of Bailing Capsule; BC-H, high dosage of Bailing Capsule. Data are expressed as mean ± SD (*n* = 8). **p < 0.01.

Similarly, it was apparent that more FASN-positive cells were present in the DN group, whereas fewer ACOX1-, PPARα-, SCD-positive cells were present in the DN group relative to the control group (*p* < 0.01, Figure 9). Compared with the DN group, Bailing Capsule group showed a significant reduction in FASN and an increase in ACOX1, PPARα, and SCD (*p* < 0.01, Figure 9).

### Effects of bailing capsule on peroxisome proliferator activated receptor pathway protein expression levels in diabetic nephropathy rats determined by western blot

**FIGURE 10:**
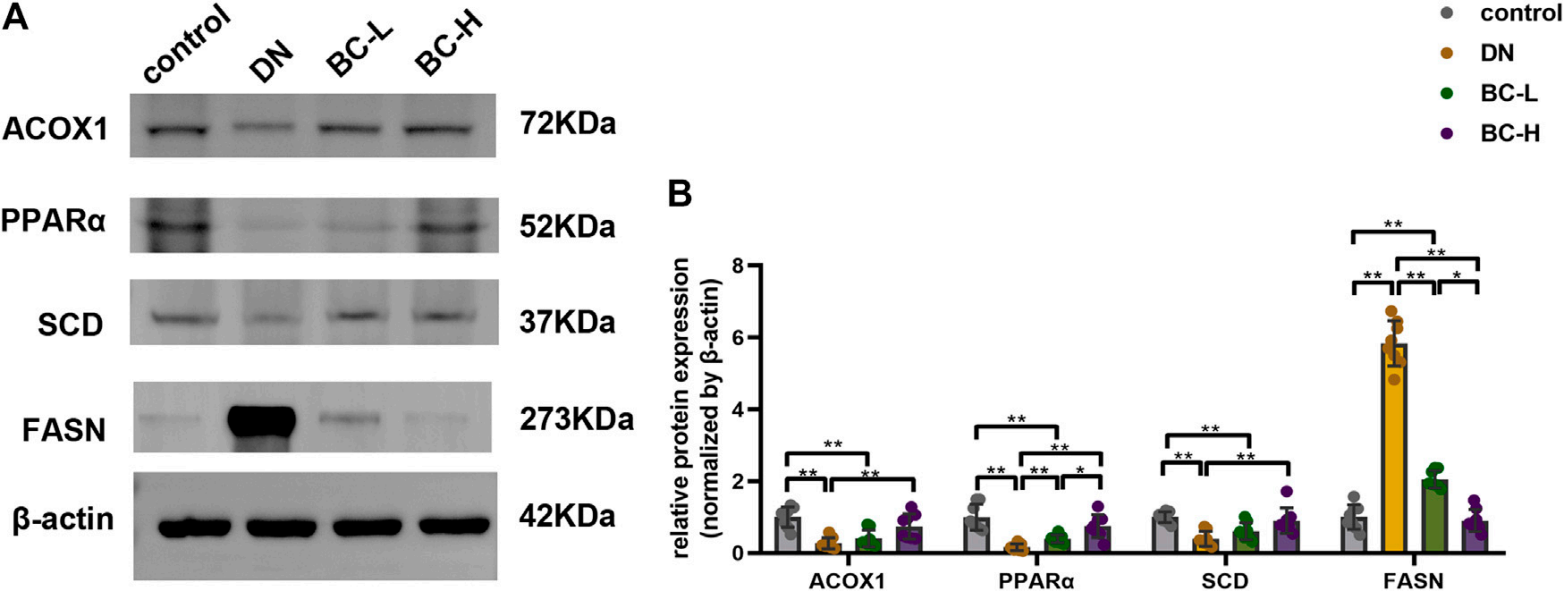
Effect of Bailing Capsule treatment on the relative protein expression levels of ACOX1, PPARα, SCD and FASN in kidneys of rats shown by western blot. **(A)** Representative immunoblots of ACOX1, PPARα, SCD, and FASN in kidneys of rats among different groups. Western blot showed that Bailing Capsule increased the protein levels of ACOX1, PPARα, SCD and inhibited the protein levels of FASN in DN rats. **(B)** Quantification of immunoblots of ACOX1, PPARα, SCD, and FASN. DN, diabetic nephropathy; BC-L, low dosage of Bailing Capsule; BC-H, high dosage of Bailing Capsule. Data are expressed as mean ± SD (*n* = 8). *p < 0.05 and **p < 0.01.

Western blotting analysis showed a significant reduction in the expression levels of ACOX1, PPARα, and SCD in the kidneys of DN rats compared to the control group (*p* < 0.01, Figure 10). The expression levels of ACOX1, PPARα, and SCD were upregulated in DN kidneys by high dosage of Bailing Capsule treatment (*p* < 0.01, Figure 10). In addition, Bailing Capsule inhibited the overexpression of FASN in DN rats (*p* < 0.01, Figure 10). Therefore, these findings showed that Bailing Capsule administration has an ameliorative effect on the PPAR pathway protein expression changes induced in the kidney by DN.

## Discussion

In this study, our data show that both low and high doses of Bailing Capsule relieves hyperglycemia. The active ingredient of Bailing Capsule is *C. sinensis*. Yang et al. found that *Cordyceps Cidadae* polysaccharides improve glucose tolerance in DN rats (Yang et al., 2020). *Hirsutella Sinensis*, an anamorph of *C. sinensis*, reduced the levels of FBG in db/db mice (Lu et al., 2019). However, Kan et al. reported that *C. sinensis* did not improve hyperglycemia in a KK/HIJ diabetic mouse model (Kan et al., 2012). The differences in these results may be due to the differences in the diabetic animal models. Regarding the renal protection effect, this study found that Bailing Capsule reduced kidney hypertrophy, serum creatinine, BUN, 24 h urinary protein, and urinary albumin, and moderated the renal pathology in diabetic rats. *C. sinensis* was previously found to improve renal function in rodent diabetic models, including STZ-induced diabetic SD rats (Yang et al., 2020), unilateral ureteral obstruction (UUO) rats (Du et al., 2015), and db/db mice (Lu et al., 2019). The mechanism involves the alleviation of the inflammatory response and modulation of gut microbiota dysbiosis via suppression of the toll-like receptor 4 (TLR4)/nuclear factor-κB (NF-κB) and TGF-β1/Smad signaling pathways (Yang et al., 2020), inhibiting Bcl-2-associated athanogene 3 (BAG3) and α-smooth muscle actin (α-SMA) in tubular epithelium (Du et al., 2015), inhibiting the p38 and extracellular regulated kinase (ERK) signaling pathways (Dong et al., 2019), reducing oxidative stress (Zhao et al., 2018) and influencing the mitogen-activated protein kinase (MAPK) signaling pathways (Zhao et al., 2018).

Among the pathogenic mechanisms of DN, the disturbance of fatty acid metabolism is an important factor leading to diabetic kidney injury (Herman-Edelstein et al., 2014; Chen et al., 2019). Prolonged hyperglycemia in diabetes aggravates fatty acid synthesis and TG accumulation. In a DN animal model, the effect of renal lipid accumulation was proven to be associated with glomerulosclerosis and tubulointerstitial injury (Jiang et al., 2007; Herman-Edelstein et al., 2014). In this study, we found Bailing Capsule reduced renal lipid deposition in DN rats, which may protect the kidney function.

Renal lipid accumulation in DN kidneys is mainly caused by the activation of fatty acid synthesis or the reduction of fatty acid oxidation. Some key enzymes catalyze fatty acid synthesis, such as FASN and acetyl-CoA carboxylase (ACC). In this study, we found marked upregulation of FASN mRNA and protein expression in DN kidney tissue. Previous studies also showed that the expression of FASN, which promotes fatty acid synthesis, is significantly increased in diabetic kidneys (Wang et al., 2005). High dosage of Bailing Capsule significantly inhibited lipid accumulation and reduced the expression levels of FASN in diabetic kidneys. SCD is a rate-limiting enzyme that converts saturated fatty acids to monounsaturated fatty acids, leading to the formation of neutral lipid droplets. In this study, the reduction in renal SCD expression by DN was alleviated by Bailing Capsule treatment. Iwai et al. found that in HFD-induced diabetic mice, renal SCD expression was significantly reduced. Overexpression of SCD reduced saturated fatty acid (SFA)-induced proximal tubular epithelial cell apoptosis. Enhancing SCD may become a promising therapeutic target to reduce SFA-induced lipotoxicity (Iwai et al., 2016). Thus, our data suggest that Bailing Capsule inhibits lipogenesis by activating SCD and suppressing FASN expression to moderate the progression of DN in rats.

In addition, in this study, we found differentially expressed kidney genes in Bailing Capsule-treated group were enriched in the PPAR pathway. In particular, Bailing Capsule treatment enhanced renal PPARα expression. PPARα is a ligand-dependent nuclear receptor that regulates lipid metabolism (Desvergne and Wahli, 1999). PPARα can be activated by exogenous compounds, such as fatty acids (Desvergne and Wahli, 1999). PPARα is mainly expressed in liver, kidney, and heart tissue, which have abundant mitochondrial and high activities of β-oxidation. The main function of PPARα is the regulation of fatty acid oxidation. In recent decades, several clinical trials demonstrated the beneficial effects of fenofibrate, a PPARα agonist, on type 2 diabetic patients, including decreases in creatinine clearance and estimated glomerular filtration rate (eGFR) (Forsblom et al., 2010; Ismail-Beigi et al., 2010; Wright and Dodson, 2011). These promising results suggest that PPARα is a potential drug target for DN treatment. Activation of PPARα was found to ameliorate kidney function in diabetic animal models through multiple effects, including reduction of renal lipotoxicity (Tanaka et al., 2011), inflammation (Shin et al., 2009), fibrosis (Hou et al., 2010) and oxidative stress (Balakumar et al., 2009). On the other hand, diabetic PPARα knockout mice have more severe kidney dysfunction symptoms, including albuminuria, glomerular sclerosis, and mesangial area expansion (Park et al., 2006). Our results showed that the expression of PPARα was reduced in DN rat kidneys. And Bailing capsule treatment increased renal PPARα expression. In addition, we found that the expression levels of PPARα target genes, such as ACOX1 were also upregulated by Bailing Capsule treatment in DN rats. ACOX1 is a lipolytic enzyme that is regulated by PPARα in lipid oxidation (Morise et al., 2009; Huang et al., 2012). In this study, we found that Bailing Capsule upregulated the expression of PPARα and ACOX1 in diabetic kidneys. These results indicated that Bailing Capsule activates PPARα pathway and downstream ACOX1 to enhance lipolysis, leading to the protection of kidney function.

The current work investigated the mechanism of Bailing Capsule moderates kidney function in DN rats. The transcriptome array is reliable and novel for searching the multiple targets and pathways affected by TCMs which have multiple active components. Furthermore, our study addressed the evidence that Bailing Capsule moderated renal lipid metabolism to moderated kidney function. However, this study has several limitations. Considering that HFD/STZ injection rats is one of DN model, other kidney disease models should be used to validate our findings. Moreover, the beginning time of treatment and duration also need to be explored.

## Conclusion

**FIGURE 11:**
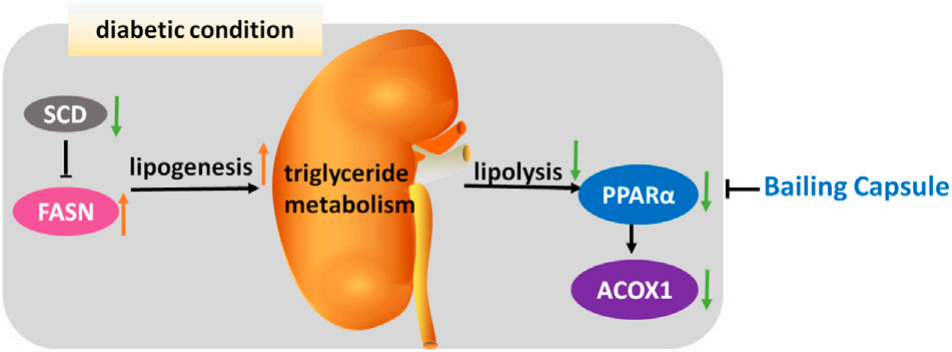
Mechanism underlying renal-protective effect of Bailing Capsule. Intracellular lipid accumulation is governed by the syntheses and oxidation of triglyceride in kidney. In diabetic condition, lipogenesis is activated by FASN, whereas lipolysis is inhibited, through PPARα and ACOX1, leading to renal lipid accumulation. Bailing capsule inhibited this triglyceride accumulation through PPAR pathway.

In conclusion, this study provides convincing evidence of the efficacy of Bailing Capsule in preventing the development and progression of DN. The effects of Bailing Capsule are apparently associated with the activation of renal lipolysis and the inhibition of renal lipogenesis, leading to a reduction in kidney lipid accumulation (Figure 11).

## Data Availability

The datasets presented in this study can be found in online repositories. The names of the repository/repositories and accession number(s) can be found below: https://www.ncbi.nlm.nih.gov/geo/query/acc.cgi?acc=GSE198021.

## References

[B1] Aihara K. Ikeda Y. Yagi S. Akaike M. Matsumoto T. (2010). Transforming growth factor-β1 as a common target molecule for development of cardiovascular diseases, renal insufficiency and metabolic syndrome. Cardiol. Res. Pract. 2011, 175381. 10.4061/2011/175381 PubMed Abstract | 10.4061/2011/175381 | Google Scholar 21234356 PMC3018616

[B2] Balakumar P. Chakkarwar V. A. Singh M. (2009). Ameliorative effect of combination of benfotiamine and fenofibrate in diabetes-induced vascular endothelial dysfunction and nephropathy in the rat. Mol. Cell. Biochem. 320, 149–162. 10.1007/s11010-008-9917-z PubMed Abstract | 10.1007/s11010-008-9917-z | Google Scholar 18830571

[B3] Baynes J. W. (1991). Role of oxidative stress in development of complications in diabetes. Diabetes 40, 405–412. 10.2337/diab.40.4.405 PubMed Abstract | 10.2337/diab.40.4.405 | Google Scholar 2010041

[B4] Chen X. Han Y. Gao P. Yang M. Xiao L. Xiong X. (2019). Disulfide-bond A oxidoreductase-like protein protects against ectopic fat deposition and lipid-related kidney damage in diabetic nephropathy. Kidney Int. 95, 880–895. 10.1016/j.kint.2018.10.038 PubMed Abstract | 10.1016/j.kint.2018.10.038 | Google Scholar 30791996

[B7] Dai B. Wang Z. Z. Zhang H. Han M. X. Zhang G. X. Chen J. W. (2020). Antihypertensive properties of a traditional Chinese medicine GAO-ZI-YAO in elderly spontaneous hypertensive rats. Biomed. Pharmacother. 131, 110739. 10.1016/j.biopha.2020.110739 PubMed Abstract | 10.1016/j.biopha.2020.110739 | Google Scholar 32932045

[B8] Danda R. S. Habiba N. M. Rincon-Choles H. Bhandari B. K. Barnes J. L. Abboud H. E. (2005). Kidney involvement in a nongenetic rat model of type 2 diabetes. Kidney Int. 68, 2562–2571. 10.1111/j.1523-1755.2005.00727.x PubMed Abstract | 10.1111/j.1523-1755.2005.00727.x | Google Scholar 16316331

[B9] Deng Y. Y. Chen Y. P. He X. L. Li L. (2001). Study of Cordyceps on mechanism in delaying chronic renal failure. Chin. J. Integr. Traditional West. Nephrol. 2, 318–323. 10.3969/j.issn.1009-587X.2001.07.004 10.3969/j.issn.1009-587X.2001.07.004 | Google Scholar

[B10] Desvergne B. Wahli W. (1999). Peroxisome proliferator-activated receptors: Nuclear control of metabolism. Endocr. Rev. 20, 649–688. 10.1210/edrv.20.5.0380 PubMed Abstract | 10.1210/edrv.20.5.0380 | Google Scholar 10529898

[B11] Ding M. Tang Z. Liu W. Shao T. Yuan P. Chen K. (2021). Burdock fructooligosaccharide attenuates high glucose-induced apoptosis and oxidative stress injury in renal tubular epithelial cells. Front. Pharmacol. 12, 784187. 10.3389/fphar.2021.784187 PubMed Abstract | 10.3389/fphar.2021.784187 | Google Scholar 34955856 PMC8695902

[B12] Dong Z. Sun Y. Wei G. Li S. Zhao Z. (2019). A nucleoside/nucleobase-rich extract from Cordyceps sinensis inhibits the epithelial-mesenchymal transition and protects against renal fibrosis in diabetic nephropathy. Molecules 24, E4119. 10.3390/molecules24224119 PubMed Abstract | 10.3390/molecules24224119 | Google Scholar 31739543 PMC6891521

[B13] Du F. Li S. Wang T. Zhang H. Y. Zong Z. H. Du Z. X. (2015). Cordyceps sinensis attenuates renal fibrosis and suppresses BAG3 induction in obstructed rat kidney. Am. J. Transl. Res. 7, 932–940. PubMed Abstract | Google Scholar 26175854 PMC4494144

[B14] Folch J. Lees M. Sloane Stanley G. H. (1957). A simple method for the isolation and purification of total lipides from animal tissues. J. Biol. Chem. 226, 497–509. 10.1016/s0021-9258(18)64849-5 PubMed Abstract | 10.1016/s0021-9258(18)64849-5 | Google Scholar 13428781

[B15] Forsblom C. Hiukka A. Leinonen E. S. Sundvall J. Groop P. H. Taskinen M. R. (2010). Effects of long-term fenofibrate treatment on markers of renal function in type 2 diabetes: The FIELD helsinki substudy. Diabetes Care 33, 215–220. 10.2337/dc09-0621 PubMed Abstract | 10.2337/dc09-0621 | Google Scholar 19846798 PMC2809252

[B16] Furman B. L. (2015). Streptozotocin-induced diabetic models in mice and rats. Curr. Protoc. Pharmacol. 70, 5.47.1–5.47.20. 10.1002/0471141755.ph0547s70 10.1002/0471141755.ph0547s70 | Google Scholar 26331889

[B17] Gross C. Belville C. Lavergne M. Choltus H. Jabaudon M. Blondonnet R. (2020). Advanced glycation end products and receptor (RAGE) promote wound healing of human corneal epithelial cells. Invest. Ophthalmol. Vis. Sci. 61, 14. 10.1167/iovs.61.3.14 PubMed Abstract | 10.1167/iovs.61.3.14 | Google Scholar PMC7401750 32176265

[B18] Guo Z. Jia J. Tu Y. Jin C. Guo C. Song F. (2021). Altered jagged1-notch1 signaling in enhanced dysfunctional neovascularization and delayed angiogenesis after ischemic stroke in HFD/STZ induced type 2 diabetes rats. Front. Physiol. 12, 687947. 10.3389/fphys.2021.687947 PubMed Abstract | 10.3389/fphys.2021.687947 | Google Scholar 34305641 PMC8297620

[B19] Herman-Edelstein M. Scherzer P. Tobar A. Levi M. Gafter U. (2014). Altered renal lipid metabolism and renal lipid accumulation in human diabetic nephropathy. J. Lipid Res. 55, 561–572. 10.1194/jlr.P040501 PubMed Abstract | 10.1194/jlr.P040501 | Google Scholar 24371263 PMC3934740

[B20] Hou X. Shen Y. H. Li C. Wang F. Zhang C. Bu P. (2010). PPARalpha agonist fenofibrate protects the kidney from hypertensive injury in spontaneously hypertensive rats via inhibition of oxidative stress and MAPK activity. Biochem. Biophys. Res. Commun. 394, 653–659. 10.1016/j.bbrc.2010.03.043 PubMed Abstract | 10.1016/j.bbrc.2010.03.043 | Google Scholar 20226762

[B21] Hu X. Wang J. Yang H. Ji S. Li Y. Xu B. (2021). Bailing Capsule combined with α-ketoacid tablets for stage 3 chronic kidney disease: Protocol of a double-blinded, randomized, controlled trial. Med. Baltim. 100, e25759. 10.1097/MD.0000000000025759 10.1097/MD.0000000000025759 | Google Scholar PMC8136994 34011035

[B22] Huang Da W. Sherman B. T. Lempicki R. A. (2009). Systematic and integrative analysis of large gene lists using DAVID bioinformatics resources. Nat. Protoc. 4, 44–57. 10.1038/nprot.2008.211 PubMed Abstract | 10.1038/nprot.2008.211 | Google Scholar 19131956

[B23] Huang J. Jia Y. Fu T. Viswakarma N. Bai L. Rao M. S. (2012). Sustained activation of PPARα by endogenous ligands increases hepatic fatty acid oxidation and prevents obesity in ob/ob mice. Faseb J. 26, 628–638. 10.1096/fj.11-194019 PubMed Abstract | 10.1096/fj.11-194019 | Google Scholar 22009939 PMC3290446

[B24] Ismail-Beigi F. Craven T. Banerji M. A. Basile J. Calles J. Cohen R. M. (2010). Effect of intensive treatment of hyperglycaemia on microvascular outcomes in type 2 diabetes: An analysis of the ACCORD randomised trial. Lancet 376, 419–430. 10.1016/S0140-6736(10)60576-4 PubMed Abstract | 10.1016/S0140-6736(10)60576-4 | Google Scholar 20594588 PMC4123233

[B25] Iwai T. Kume S. Chin-Kanasaki M. Kuwagata S. Araki H. Takeda N. (2016). Stearoyl-CoA desaturase-1 protects cells against lipotoxicity-mediated apoptosis in proximal tubular cells. Int. J. Mol. Sci. 17, E1868. 10.3390/ijms17111868 PubMed Abstract | 10.3390/ijms17111868 | Google Scholar 27834856 PMC5133868

[B26] Jiang T. Wang X. X. Scherzer P. Wilson P. Tallman J. Takahashi H. (2007). Farnesoid X receptor modulates renal lipid metabolism, fibrosis, and diabetic nephropathy. Diabetes 56, 2485–2493. 10.2337/db06-1642 PubMed Abstract | 10.2337/db06-1642 | Google Scholar 17660268

[B27] Kan W. C. Wang H. Y. Chien C. C. Li S. L. Chen Y. C. Chang L. H. (2012). Effects of extract from solid-state fermented Cordyceps sinensis on type 2 diabetes mellitus. Evid. Based. Complement. Altern. Med. 2012, 743107. 10.1155/2012/743107 PubMed Abstract | 10.1155/2012/743107 | Google Scholar PMC3296307 22474523

[B28] Lapolla A. Traldi P. Fedele D. (2005). Importance of measuring products of non-enzymatic glycation of proteins. Clin. Biochem. 38, 103–115. 10.1016/j.clinbiochem.2004.09.007 PubMed Abstract | 10.1016/j.clinbiochem.2004.09.007 | Google Scholar 15642271

[B29] Liu Y. Huang H. Gao R. Liu Y. (2020). Dynamic phenotypes and molecular mechanisms to understand the pathogenesis of diabetic nephropathy in two widely used animal models of type 2 diabetes mellitus. Front. Cell Dev. Biol. 8, 172. 10.3389/fcell.2020.00172 PubMed Abstract | 10.3389/fcell.2020.00172 | Google Scholar 32266256 PMC7098383

[B30] Logan S. Jiang C. Yan Y. Inagaki Y. Arzua T. Bai X. (2018). Propofol alters long non-coding RNA profiles in the neonatal mouse Hippocampus: Implication of novel mechanisms in anesthetic-induced developmental neurotoxicity. Cell. Physiol. biochem. 49, 2496–2510. 10.1159/000493875 PubMed Abstract | 10.1159/000493875 | Google Scholar 30261491 PMC6221186

[B31] Lu Z. Li S. Sun R. Jia X. Xu C. Aa J. (2019). Hirsutella sinensis treatment shows protective effects on renal injury and metabolic modulation in db/db mice. Evid. Based. Complement. Altern. Med. 2019, 4732858. 10.1155/2019/4732858 PubMed Abstract | 10.1155/2019/4732858 | Google Scholar PMC6475559 31080482

[B32] Luc K. Schramm-Luc A. Guzik T. J. Mikolajczyk T. P. (2019). Oxidative stress and inflammatory markers in prediabetes and diabetes. J. Physiol. Pharmacol. 70. 10.26402/jpp.2019.6.01 PubMed Abstract | 10.26402/jpp.2019.6.01 | Google Scholar 32084643

[B53] Ma X. Yan W. (2021). Effects of bailing capsule combined with valsartan on renal protection and cellular immune function in patients with chronic glomerulonephritis. Chin. Archives Traditional Chin. Med. 39, 220–223. 10.13193/j.issn.1673-7717.2021.09.055 10.13193/j.issn.1673-7717.2021.09.055 | Google Scholar

[B33] Mehlem A. Hagberg C. E. Muhl L. Eriksson U. Falkevall A. (2013). Imaging of neutral lipids by oil red O for analyzing the metabolic status in health and disease. Nat. Protoc. 8, 1149–1154. 10.1038/nprot.2013.055 PubMed Abstract | 10.1038/nprot.2013.055 | Google Scholar 23702831

[B34] Morise A. Thomas C. Landrier J. F. Besnard P. Hermier D. (2009). Hepatic lipid metabolism response to dietary fatty acids is differently modulated by PPARalpha in male and female mice. Eur. J. Nutr. 48, 465–473. 10.1007/s00394-009-0037-7 PubMed Abstract | 10.1007/s00394-009-0037-7 | Google Scholar 19588182

[B35] Nath S. Ghosh S. K. Choudhury Y. (2017). A murine model of type 2 diabetes mellitus developed using a combination of high fat diet and multiple low doses of streptozotocin treatment mimics the metabolic characteristics of type 2 diabetes mellitus in humans. J. Pharmacol. Toxicol. Methods 84, 20–30. 10.1016/j.vascn.2016.10.007 PubMed Abstract | 10.1016/j.vascn.2016.10.007 | Google Scholar 27773844

[B6] Niu C. Li H. (2019). Effect of Bailing capsule combined with simvastatin on renal function and oxidative stress in patients with diabeitc nephropathy. Foreine Mecical Sci. Sction Medgeogr. 40, 275–276. 10.3969/j.issn.1001-8883.2019.03.018 10.3969/j.issn.1001-8883.2019.03.018 | Google Scholar

[B36] Park C. W. Kim H. W. Ko S. H. Chung H. W. Lim S. W. Yang C. W. (2006). Accelerated diabetic nephropathy in mice lacking the peroxisome proliferator-activated receptor alpha. Diabetes 55, 885–893. 10.2337/diabetes.55.04.06.db05-1329 PubMed Abstract | 10.2337/diabetes.55.04.06.db05-1329 | Google Scholar 16567507

[B37] Ren H. J. Sun Y. L. Yuan B. (2019). Chinese patent medicine bailing capsule for treating lupus nephritis: A protocol for systematic review and meta-analysis. Med. Baltim. 98, e17041. 10.1097/MD.0000000000017041 PubMed Abstract | 10.1097/MD.0000000000017041 | Google Scholar PMC6750295 31517824

[B38] Said E. Zaitone S. A. Eldosoky M. Elsherbiny N. M. (2018). Nifuroxazide, a STAT3 inhibitor, mitigates inflammatory burden and protects against diabetes-induced nephropathy in rats. Chem. Biol. Interact. 281, 111–120. 10.1016/j.cbi.2017.12.030 PubMed Abstract | 10.1016/j.cbi.2017.12.030 | Google Scholar 29291386

[B39] Sheng X. Dong Y. Cheng D. Wang N. Guo Y. (2020). Efficacy and safety of bailing capsules in the treatment of type 2 diabetic nephropathy: A meta-analysis. Ann. Palliat. Med. 9, 3885–3898. 10.21037/apm-20-1799 PubMed Abstract | 10.21037/apm-20-1799 | Google Scholar 33222468

[B40] Shin S. J. Lim J. H. Chung S. Youn D. Y. Chung H. W. Kim H. W. (2009). Peroxisome proliferator-activated receptor-alpha activator fenofibrate prevents high-fat diet-induced renal lipotoxicity in spontaneously hypertensive rats. Hypertens. Res. 32, 835–845. 10.1038/hr.2009.107 PubMed Abstract | 10.1038/hr.2009.107 | Google Scholar 19644507

[B41] Sun H. Saeedi P. Karuranga S. Pinkepank M. Ogurtsova K. Duncan B. B. (2022). IDF Diabetes Atlas: Global, regional and country-level diabetes prevalence estimates for 2021 and projections for 2045. Diabetes Res. Clin. Pract. 183, 109119. 10.1016/j.diabres.2021.109119 PubMed Abstract | 10.1016/j.diabres.2021.109119 | Google Scholar 34879977 PMC11057359

[B42] Tanaka Y. Kume S. Araki S. Isshiki K. Chin-Kanasaki M. Sakaguchi M. (2011). Fenofibrate, a PPARα agonist, has renoprotective effects in mice by enhancing renal lipolysis. Kidney Int. 79, 871–882. 10.1038/ki.2010.530 PubMed Abstract | 10.1038/ki.2010.530 | Google Scholar 21270762

[B43] Wang D. Zhang G. Chen X. Wei T. Liu C. Chen C. (2018). Sitagliptin ameliorates diabetic nephropathy by blocking TGF-β1/Smad signaling pathway. Int. J. Mol. Med. 41, 2784–2792. 10.3892/ijmm.2018.3504 PubMed Abstract | 10.3892/ijmm.2018.3504 | Google Scholar 29484381 PMC5846674

[B44] Wang W. Zhang X. N. Yin H. Li X. B. Hu X. P. Liu H. (2013). Effects of bailing capsules for renal transplant recipients: A retrospective clinical study. Chin. Med. J. 126, 1895–1899. 10.3760/cma.j.issn.0366-6999.20130483 PubMed Abstract | 10.3760/cma.j.issn.0366-6999.20130483 | Google Scholar 23673106

[B45] Wang Z. Jiang T. Li J. Proctor G. Mcmanaman J. L. Lucia S. (2005). Regulation of renal lipid metabolism, lipid accumulation, and glomerulosclerosis in FVBdb/db mice with type 2 diabetes. Diabetes 54, 2328–2335. 10.2337/diabetes.54.8.2328 PubMed Abstract | 10.2337/diabetes.54.8.2328 | Google Scholar 16046298

[B46] Wright A. D. Dodson P. M. (2011). Medical management of diabetic retinopathy: Fenofibrate and ACCORD eye studies. Eye (Lond) 25, 843–849. 10.1038/eye.2011.62 PubMed Abstract | 10.1038/eye.2011.62 | Google Scholar 21436845 PMC3178166

[B47] Wu J. Wei Z. Cheng P. Qian C. Xu F. Yang Y. (2020). Rhein modulates host purine metabolism in intestine through gut microbiota and ameliorates experimental colitis. Theranostics 10, 10665–10679. 10.7150/thno.43528 PubMed Abstract | 10.7150/thno.43528 | Google Scholar 32929373 PMC7482825

[B49] Xiao C. Xiao P. Li X. Li X. Li H. Chen Y. (2018). Cordyceps sinensis may inhibit Th22 cell chemotaxis to improve kidney function in lgA nephropathy. Am. J. Transl. Res. 10, 857–865. PubMed Abstract | Google Scholar 29636875 PMC5883126

[B50] Xiao L. Xu X. Zhang F. Wang M. Xu Y. Tang D. (2017). The mitochondria-targeted antioxidant MitoQ ameliorated tubular injury mediated by mitophagy in diabetic kidney disease via Nrf2/PINK1. Redox Biol. 11, 297–311. 10.1016/j.redox.2016.12.022 PubMed Abstract | 10.1016/j.redox.2016.12.022 | Google Scholar 28033563 PMC5196243

[B51] Xu H. Li X. Yuan X. Yuan Q. Chen W. Peng Z. (2020). A meta-analysis of the clinical efficacy and safety of Bailing capsules in the treatment of nephrotic syndrome. Ann. Palliat. Med. 9, 3170–3181. 10.21037/apm-20-1252 PubMed Abstract | 10.21037/apm-20-1252 | Google Scholar 32921102

[B52] Xu Y. Bai L. Chen X. Li Y. Qin Y. Meng X. (2018). 6-Shogaol ameliorates diabetic nephropathy through anti-inflammatory, hyperlipidemic, anti-oxidative activity in db/db mice. Biomed. Pharmacother. 97, 633–641. 10.1016/j.biopha.2017.10.084 PubMed Abstract | 10.1016/j.biopha.2017.10.084 | Google Scholar 29101807

[B54] Yang J. Dong H. Wang Y. Jiang Y. Zhang W. Lu Y. (2020). Cordyceps cicadae polysaccharides ameliorated renal interstitial fibrosis in diabetic nephropathy rats by repressing inflammation and modulating gut microbiota dysbiosis. Int. J. Biol. Macromol. 163, 442–456. 10.1016/j.ijbiomac.2020.06.153 PubMed Abstract | 10.1016/j.ijbiomac.2020.06.153 | Google Scholar 32592781

[B55] Yu W. Duan S. Yu Z. (2021). The effect of Bailing capsules combined with losartan to treat diabetic glomerulosclerosis and the combination's effect on blood and urine biochemistry. Am. J. Transl. Res. 13, 6873–6880. PubMed Abstract | Google Scholar 34306438 PMC8290721

[B56] Zhang Q. Xiao X. Zheng J. Li M. Yu M. Ping F. (2019). Shenqi jiangtang granule ameliorates kidney function by inhibiting apoptosis in a diabetic rat model. Evid. Based. Complement. Altern. Med. 2019, 3240618. 10.1155/2019/3240618 PubMed Abstract | 10.1155/2019/3240618 | Google Scholar PMC6886351 31827549

[B57] Zhao K. Gao Q. Zong C. Ge L. Liu J. (2018). Cordyceps sinensis prevents contrast-induced nephropathy in diabetic rats: Its underlying mechanism. Int. J. Clin. Exp. Pathol. 11, 5571–5580. PubMed Abstract | Google Scholar 31949644 PMC6963074

[B58] Zimmet P. Alberti K. G. Magliano D. J. Bennett P. H. (2016). Diabetes mellitus statistics on prevalence and mortality: Facts and fallacies. Nat. Rev. Endocrinol. 12, 616–622. 10.1038/nrendo.2016.105 PubMed Abstract | 10.1038/nrendo.2016.105 | Google Scholar 27388988

